# Analysis of the molecular composition of humic substances and their effects on physiological metabolism in maize based on untargeted metabolomics

**DOI:** 10.3389/fpls.2023.1122621

**Published:** 2023-05-22

**Authors:** Yuhong Wang, Yanli Lu, Lei Wang, Guipei Song, Lu Ni, Mengze Xu, Caie Nie, Baoguo Li, Youlu Bai

**Affiliations:** ^1^ State Key Laboratory of Efficient Utilization of Arid and Semi-arid Arable Land in Northern China/Key Laboratory of Plant Nutrition and Fertilizer, Ministry of Agriculture and Rural Affairs/Institute of Agricultural Resources and Regional Planning, Chinese Academy of Agricultural Sciences, Beijing, China; ^2^ College of Land Science and Technology, China Agricultural University, Beijing, China

**Keywords:** humic substances, metabolomics, hormone-like activity, starch and sucrose metabolism, chemical structures

## Abstract

**Introduction:**

Humic substances (HSs), components of plant biostimulants, are known to influence plant physiological processes, nutrient uptake and plant growth, thereby increasing crop yield. However, few studies have focused on the impact of HS on overall plant metabolism, and there is still debate over the connection between HS’ structural characteristics and their stimulatory actions.

**Methods:**

In this study, two different HSs (AHA, Aojia humic acid and SHA, Shandong humic acid) screened in a previous experiment were chosen for foliar spraying, and plant samples were collected on the tenth day after spraying (62 days after germination) to investigate the effects of different HSs on photosynthesis, dry matter accumulation, carbon and nitrogen metabolism and overall metabolism in maize leaf.

**Results and discussion:**

The results showed different molecular compositions for AHA and SHA and a total of 510 small molecules with significant differences were screened using an ESI-OPLC-MS techno. AHA and SHA exerted different effects on maize growth, with the AHA inducing more effective stimulation than the SHA doing. Untargeted metabolomic analysis revealed that the phospholipid components of maize leaves treated by SHA generally increased significantly than that in the AHA and control treatments. Additionally, both HS-treated maize leaves exhibited different levels of accumulation of trans-zeatin, but SHA treatment significantly decreased the accumulation of zeatin riboside. Compared to CK treatment, AHA treatment resulted in the reorganization of four metabolic pathways: starch and sucrose metabolism, TCA cycle, stilbenes, diarylheptanes, and curcumin biosynthesis, and ABC transport, SHA treatment modified starch and sucrose metabolism and unsaturated fatty acid biosynthesis. These results demonstrate that HSs exert their function through a multifaceted mechanism of action, partially connected to their hormone-like activity but also involving hormoneindependent signaling pathways.

## 1 Introduction

An inventive approach to the problems facing sustainable agriculture is the use of natural plant biostimulants. Humic substances (HSs), one type of plant biostimulants, has received increasing attention from researchers and farmers. Numerous studies have shown that plants grown in soils with sufficient HSs or exposed to foliar sprays composed of HSs are healthier because these plants are well adapted to and resistant to stressful conditions and show increased yield through increasing nutrient uptake (Quaggiotti et al., 2004; Mora et al., 2010).

For many years, soil scientists have been working to determine the chemical characteristics and molecular structure of HSs and to study how they regulate plant growth and development. Several studies have proposed the hypothesis that HSs may act on plants through two different mechanisms: (1) indirectly, by enhancing the chemical, physical and biological qualities of the soil; and (2) directly, by regulating growth processes, nutrient transport systems and primary and secondary metabolism through HSs active components (Canellas and Olivares, 2014; Nardi et al., 2016; Nunes et al., 2019; Pizzeghello et al., 2020). For example, HSs stimulate the synthesis of plasma membrane H^+^-ATPase. Through the combination of ATP hydrolysis and H^+^ transfer across the cell membrane, this enzyme promotes root growth by acidifying the plasma outer body, loosening the cell wall, and lengthening the cell (Zandonadi et al., 2007; Canellas and Olivares, 2014; De Azevedo et al., 2019). Activation of plasma membrane H^+^-ATPase also improves plant nutrition by increasing the electrochemical proton gradient that drives ion transport across the cell membrane (Canellas et al., 2002). Earthworm-like HSs induce changes in the metabolism of reactive oxygen species (ROS) in plants after root application (García et al., 2012; García et al., 2014). ROS have been categorized as signal transduction molecules that participate in transduction mechanisms regulating metabolic activities, including plant growth and development, although they are mainly considered as hazardous compounds resulting from aerobic metabolism (Mittler et al., 2011; Mittler, 2017). In this context, ROS has been proposed as a possible target for the action of HSs in plants (Garcia et al., 2016; Roomi et al., 2018). Additionally, Zandonadi et al. (2010) showed that HSs induce nitric oxide (NO) production at the site of lateral root emergence. Both primary and secondary metabolism have been documented to be altered by HSs. For example, Nardi et al. (2007) observed the upregulation of glycolysis and the tricarboxylic acid cycle (TCA) enzymes in maize plants treated with 1 mg C L^-1^ HS, which also increased nitrogen uptake/assimilation and nitrogen metabolism. Furthermore, HS treatment was able to lower the pH of the root surface, promote H^+^/ ${\;}_{\text{NO}}^{3}{\;}^{-}$ symport and stimulate nitrate uptake, transport and nitrogen metabolism enzyme activity. In addition, HS treatment exerted a positive effect on phenylpropane metabolism and enzymes associated with cytoprotection (Garcia et al., 2016). Taken together, these findings illustrate the complexity of the relationship between HSs and plant physiology and emphasize the value of molecular approaches in understanding the nature of this interaction.

In the last decade, new molecular “omics” techniques (genomics, transcriptomics, proteomics and metabolism) have been applied to evaluate the effects of HSs on plant metabolism (Carletti et al., 2008; Nephali et al., 2020). Recent studies have described the impeccable ability and potential of omics techniques to reveal the mechanisms describing the interaction of biostimulants with plants. Trevisan et al. (2011) performed a transcriptomic analysis and Gene Ontology classification on *Arabidopsis* after three days of treatment and observed a widespread but slight modulation of the transcriptional activity involving the plant’s main metabolic functions: respiration and photosynthesis, general cellular metabolism, fatty acids, nitrogen/sulfur, plant hormones, plant development, senescence, stress response, ion and water transport. Nunes et al. (2019) used a label-free quantitative proteomic approach to analyze the effect of humic acid on the soluble protein fraction of maize seedling roots, and differences were detected in root proteins relation to energy metabolism, the cytoskeleton, cellular trafficking, protein conformation and degradation, and DNA replication. However, few studies have investigated the effects of HSs on plant metabolism via metabolomics analysis. Metabolomics is a multidisciplinary omics science that provides unique opportunities to predict the mode of action of biostimulants on crops and to identify markers of biostimulatory effects (Nephali et al., 2020). In one of the very few cases when metabolomics techniques were utilized in HS response research, Marino et al. (2013) showed that HS-treated pears and papaya calli produced more asparagine than controls using ^1^H HR-MAS NMR analysis.

Numerous studies have highlighted how HSs might improve plant responses and growth metrics, but the new challenge is to determine which parts or specific components of HSs are most likely to induce a positive response. For this analysis, the structure and composition of humic substances must be examined and characterized. In general, the characterization of HSs is divided into elemental, functional group and molecular weight categories, but these characterizations do not provide information at the molecular level. In 2001, Piccolo (2001) redefined fulvic acid (FA) as a hydrophilic small molecule conjugate with sufficient acid functional groups to allow its dispersion in solution at any pH. At neutral or alkaline pH, HSs are not stable polymers but supramolecular associations of relatively small heterogeneous molecules (polymethylenic chains, fatty acids and steroid compounds) held together by weak dispersion forces, such as van der Waals, π-π, CH-π, and interactions. As intermolecular hydrogen bonds are gradually formed at lower pH values, their conformation gradually increases until flocculation, and the concept of “humeomics” was thus introduced (Piccolo, 2001; Nebbioso and Piccolo, 2011). Furthermore, researchers have speculated that the progressive breakdown of the intra- and intermolecular interactions that support complex superstructures may reduce the complexity of humic supramolecules, releasing individual humic substances molecules that enter the plant or engage with receptors that interact with plant cells. These molecules are separated and identified by combining advanced analytical techniques (Nebbioso and Piccolo, 2011; Drosos et al., 2017). Further studies have noted that the main molecular components of HSs are fatty acids, ethers, esters, alcohols, aromatic lignified fragments, polysaccharides and peptides (Scaglia et al., 2016; Drosos et al., 2017).

The complexity of the HS structure and its associated biological activity in plants has been extensively described. Despite the fact that several approaches have been employed in research on this topic, the direct relationship between the chemical structures of HSs and their effects on plant metabolism has not yet been fully elucidated so far. Initially, the molecule size, hydrophilicity, and particular functional groups of HSs were proposed to be closely associated with their activity. According to Nardi et al. (2007), the size exclusion chromatography fraction of HSs with the lowest molecular weight is the most effective in promoting plant metabolism, the most hydrophilic, and contains more sugars and less lignin-derived materials. Similarly, the effects of different molecular size fractions of HSs on root growth have been explored (Dobbss et al., 2010; Canellas et al., 2012). While all HS induced root growth in *Arabidopsis* and maize seedlings, the intensity of their effects varied depending on HS molecular size and plant species. Further analysis revealed that the hydrophobicity index (HB/HI) of HSs obtained using NMR parameters correlates with the appearance of lateral root hairs, but that the hydrophobic carbon content correlates negatively with the induction of lateral roots (Canellas et al., 2012). Other studies have also discovered a connection between hydrophobicity and plant responses, whereby more hydrophobic humic acids showed the greatest activity in stimulating plant responses, with certain HSs lengthening roots and others increasing root density. According to Scaglia et al. (2016), the hormone-like activity of HSs extracted from compost prepared from cow manure and leather waste varies depending on the maturity stage, and the molecules correlating with growth hormone-like activity were identified as carboxylic acids and amino acids.

The combination of a fine chemical analysis of HS molecules and overall metabolic studies of their effects on plants provides a new opportunity to expand knowledge of both the detailed chemistry and use management aspects of HSs. Based on this hypothesis, we sought to analyze the molecular composition of HSs through UHPLC-QTOF-MS technology, we employed a method based on UPHLC-QTOF-MS non-target metabolome to study the overall effect on HSs on maize metabolism. The mains aim of this study were as follows: i) assaying differences in chemical composition and biological activity from two different reservoirs; ii) appraising differential effects of these HSs on altering maize growth and metabolism over a long period; iii) attempt to analyze the molecular compositions of HSs, and explore the mechanism of promoting growth from the perspective of metabolomics in maize.

## 2 Materials and methods

### 2.1 Materials

The two HSs selected for this investigation were those that had been screened in earlier corn field trials and displayed the greatest variation in effects (data not shown). One of the humic substances (AHA, Aojia humic acid) was supplied by Beijing Aojia Fertilizer Co., Ltd. (Beijing, China), using Heilongjiang lignite as the raw material, and the other (SHA, Shangdong humic acid) was supplied by Shandong Nongda Fertilizer Technology Co., Ltd. (Jinan, China), also using Inner Mongolia lignite as the raw material.

The experiment was conducted in a glass greenhouse of the Institute of Agricultural Resources and Regional Planning, Chinese Academy of Agricultural Sciences. Both plant groups were grown in pots containing a mixture of perlite/vermiculite (1:1, v/v) in glass house. The composition of the Hoagland nutrient solution is as follows: 2 mM MgSO_4_, 1 mM NH_4_NO_3_, 5 mM KNO_3_,1 mM KH_2_PO_4_, 0.005 mM KI, 5.76 mM Ca(NO_3_)_2_, 0.1 mM H_3_BO_3_, 0.15 mM MnSO_4_, 0.05 mM ZnSO_4_, 0.00016 mM CuSO_4_, 0.0019 mM CoCl_2_, 0.1 mM NaFe-EDTA. The initial pH was adjusted to 5.8 ± 0.1.

Maize seeds (*Zea mays L*., hybrid Zhengdan 958) were soaked in 0.5% NaClO for 30 min, then rinsed and soaked in water for 12 h for surface sterilization. Afterward, the seeds were sown in vermiculite-filled seedling trays and allowed to germinate in an incubator with 14 h of light at 28°C and 10 h of darkness at 20°C. The seedlings with the same growth vigor were selected and placed in pots after 7 days. During the rejuvenation period, Hoagland nutrient solution with a half-ionic strength was used. After 7 days, it was replaced with complete nutrient solution, and 1L of nutrient solution was applied every week.

### 2.2 Determination of molecular composition of HSs

HS molecular composition was determined using UHPLC-QTOF-MS as described below.

#### 2.2.1 Sample preparation

The metabolites were extracted by adding 1 mL of pre-chilled extraction solution (2:2:1, (v/v/v) methanol: acetonitrile: water solvent mixture) to 80 mg of the samples. The sample was vortexed and mixed, sonicated at low temperature for 30 min, and then centrifuged at 14000×g for 20 min at 4°C. The supernatant was dried in a vacuum centrifuge, 100 μL of aqueous acetonitrile (1:1, (v/v) acetonitrile: water) was added before the LC-MS analysis, vortexed and centrifuged at 14000×g for 15 min at 4°C, and the supernatant was collect as the sample for analysis. In addition, pooled quality control (QC) samples were prepared by combining of equal amounts of the samples.

#### 2.2.2 Liquid phase parameters

All samples were analyzed using the UHPLC (1290 Infinity LC, Agilent Technologies) system. An ACQUITY UPLC BEH HILIC column (100 mm × 2.1 mm, 1.7 µm, Waters, Ireland) was used for separation at a column oven temperature maintained at 25°C. The flow rate was set to 0.5 mL/min, while the mobile phase comprised solvent A (water, 25 mM ammonium acetate and 25 mM ammonium hydroxide) and solvent B (Acetonitrile),. Gradient elution conditions were set as follows: 0–0.5 min, 95% B; 0.5–7 min, 95–65% B; 7–8 min, 65–40% B; 8–9 min, 40% B; 9–9.1 min, 40–95% B; and 9.1–12 min, 95% B. The injection volume for each sample was 42 μL. A random order was used for continuous analysis of the samples to avoid fluctuations detection signal in the instrument. QC samples were inserted in the sample queue for monitoring and evaluating the stability of the system and the reliability of the experimental data.

#### 2.2.3 Mass spectrometry parameters

Samples eluted from the column were detected using a TripleTOF6600 high-resolution tandem mass spectrometer with Q-TOF operated in both positive and negative ion modes. The Ion spray voltage floating was wet to 5500 and -5500 V for the positive and negative ion modes, respectively. Mass spectrometry data were acquired on the information dependent acquisition IDA mode, with a TOF mass range set from 25 to 1000 Da, and the accumulation time for product ion scan was set to 0.05 s/spectra.

#### 2.2.4 Data processing

The raw MS data were converted to MzXML files by ProteoWizard MSConvert, and then peak alignment and integration were conducted using XCMS software. The parameters of XCMS were as follows: mass range 25–1000 m/z, mass tolerance 10 ppm, retention time (RT) range 0.5–12.0 min, and RT width threshold 0.2 min. In the extracted ion features, only the variables with more than 50% of the nonzero measurement values in at least one group were retained. Compound identification of metabolites was performed by comparing of accuracy m/z value (<10 ppm), and MS/MS spectra with an in-house database established with available authentic standards.

After data normalization, the processed data matrix was submitted to R package (ropls) for multivariate statistical analysis. Principal component analysis (PCA) was conducted to observe the clustering pattern of all samples and the repeatability of the intra-group samples. Orthogonal partial least squares discriminant analysis (OPLS-DA) was further applied to filter out the noise irrelevant to the classification information and improve the parsing ability and effectiveness of the model. Variables with variable importance for the projection (VIP) values from the OPLS-DA analysis larger than 1.0 were considered as potential biomarkers since they can discriminate between two compared groups.

### 2.3 Experimental design

The experiment was arranged in randomized blocks using three treatments and ten replicates. The treatments were as follows: 1) foliar application of pure water, (CK), 2) foliar application of AHA (AHA), 3) foliar application of SHA (SHA). HSs was sprayed at a concentration of 0.5%, which was selected based on supplier recommendations and preliminary experiments assessing the effects of HS spraying on maize growth. Plants underwent foliar spraying around 16:00 using a portable sprayer, with which the upper and the lower surface of leaves were treated. The first spraying was conducted at 14 days after transplantation, and the remaining foliar sprays were applied at 10-day intervals for 5 total applications. The sampling point for plant samples was 10 days after the last spraying. For non-target metabolome analysis, the tip, middle, and base of the latest fully expanded leaves of six plants were collected and mixed, and immediately frozen in liquid nitrogen and stored at -80°C for subsequent analysis. For the biochemical analysis and to measure the fresh and dry weight of each plant’s leaf, stem, and root, four samples were collected. Then, the different parts of the maize plant were oven-dried at 105°C for 30 min and maintained at 75°C for 48 h to obtain a stable dry weight. Dry matter was analyzed for total nitrogen, phosphorus and potassium percentages. Total nutrition absorption per plant was calculated based on the tissue nutrition content and dry weight.

### 2.4 Phenotypic analysis

A ruler was used to measure plant height from the stem base to the tip of the latest fully expanded leaf. Plant stem diameter was measured with caliper. Expanded leaves area was estimated non-destructively using the Yanxin-1242 portable leaf area meter of each leaf with ligule emergence. Vegetative developmental stage was based on the number of leaves with an emerged ligule and the number of visible leaves at harvest.

### 2.5 Parameters for measuring photosynthesis

The photosynthetic rates were measured between 9:00 and 11:00 using a portable photosynthesis system (Li-6400, LICOR Biosciences, Lincoln, USA). The air temperature, CO_2_ concentration and photosynthetic photon flux density in the leaf chamber were set to 28°C, 500 µmol·mol^−1^, 1500 µmol m^−2^ s^−1^, respectively. Three biological repeats were included per data point. The instantaneous WUE was calculated as WUE = Pn/Tr (Chen et al., 2018).

Relative chlorophyll concentration in leaves was recorded using a portable SPAD meter (SPAD-502 Plus, KONICA MINOLTA, Japan) with 10 reading data. Both of these values were detected using the youngest leaf with ligule emergence.

### 2.6 Measurement of physiological parameters

Fresh leaf samples, which were previously stored at −80°C, were ground to a homogenous powder with liquid nitrogen. The protein concentration was determined using the Bradford method and a UV/Vis spectrophotometer at 595 nm wavelength. The protein concentration was reported as mg of protein per g of fresh leaf. The soluble sugar concentration was calculated using the sulfuric acid-anthrone colorimetric method. The free amino acid content was determined using the ninhydrin method with leucine as the standard.

Phenylalanine ammonia-lyase (PAL) was extracted by homogenizing frozen tissue into pre-cooled sodium borate buffer (100 mM, pH 8.7) at a ratio of 1:9 (w/v). After centrifugation at 10,000 rpm/min for 10 min at 4°C, the supernatant was collected for the enzyme assay using a previously described method (Assis et al., 2001). One unit (U) of PAL activity was defined as the amount of enzyme extract producing an increase in the optical density of 0.1 per min, which was measured at 290 nm by using UV/Vis spectrophotometer. Sucrose synthase (SS) and nitrate reductase (NR) activities were measured using the corresponding assay kits according to the manufacturer’s instructions (Beijing Solarbio Technology Co., Ltd., Beijing, China). Briefly, 0.1 g leaf sample was added to 1 mL extraction solution, followed by the ice bath homogenization, and the mixture was centrifuged at 8000 g for 10 min at 4°C, supernatant was collected for further analysis. The absorbance at 480 nm was used for the calculation of SS activity and one unit of SS activity was defined as 1 µg of sucrose per g of tissue catalyzed per minute. For NR activity, 0.1 g leaf samples were extracted in 1 ml extraction solution and the mixture was centrifuged at 4000 g for 10 min. The resulting supernatant was collected for further analysis. The absorbance at 340 nm was used for the calculation of NR activity, one unit (U) of NR activity was defined as the amount of 1 μmol NADH consumed per hour per g of sample.

### 2.7 Metabolomics analysis of maize leaf

#### 2.7.1 Sample preparation

The maize leaf samples were quickly frozen in liquid nitrogen and grounded with a homogenizer. The metabolites were extracted by adding 1 mL of pre-chilled extraction solution (2:2:1, (v/v/v) methanol: acetonitrile: water-water solvent mixture) to 80 mg (fresh weight) of the samples. The sample was vortexed and mixed, sonicated at low temperature for 30 min, and then centrifuged at 14000×g for 20 min at 4°C. The supernatant was dried in vacuum centrifuge, and 100 μL of aqueous acetonitrile (1:1, (v/v) acetonitrile: water) were added before the LC-MS analysis. The samples were vortexed and centrifuged at 14000×g for 15 min at 4°C, and the supernatant was collected as the sample for analysis.

#### 2.7.2 Liquid phase parameters

The samples were separated using a UHPLC (1290 Infinity LC, Agilent Technologies) system with a C-18 column. The column temperature was 40°C. The flow rate was set to 0.4 mL/min; and the injection volume was 2 μL. The mobile phase A consisted of 25 mM ammonium acetate and 0.5% formic acid in water, mobile phase B was methanol. Gradient elution conditions were set as follows: 0–0.5 min, 5% B; 0.5–10 min, 5–100% B; 10–12 min, 100% B; 12–12.1 min, 100–5% B; 12.1–16 min, 5% B. During the whole analysis, the sample was placed in an automatic sampler at 4°C.

The mass spectrometry parameters and data processing were the same as the determination of the HS molecular composition described in Section 2.2.

### 2.8 Data analysis

Analysis of variance (one-way ANOVA) was performed using the SPSS 23 (IBM Corp) software with type of treatment as factor followed by a pairwise *post hoc* analyses (Duncan test) to determine which means differed significantly at *p* < 0.05.

## 3 Results

### 3.1 The molecular composition of HSs

**Figure 1:**
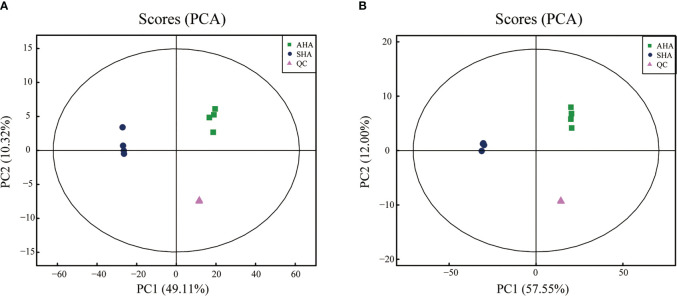
PCA score plots of the molecules profiles in positive ions mode **(A)** and negative ions mode **(B)**.

An untargeted metabolomics approach based on UHPLC-QTOF-MS was used to obtain the molecular composition profiles of these two HSs, and 1320 small molecules were detected, of which 754 were detected in positive ion mode and 566 were detected in negative ion mode. The principal component analysis (PCA) explained 59.43%, 69.55% of the overall variance in molecules detected in positive and negative ion mode, respectively (**Figure 1**). The PCA score plots (**Figure 1**) showed two main clusters accounting for the AHA and SHA, respectively. Within each cluster, the molecular composition profiles did not overlap, thus indicating distinct molecular signatures.

**Figure 2:**
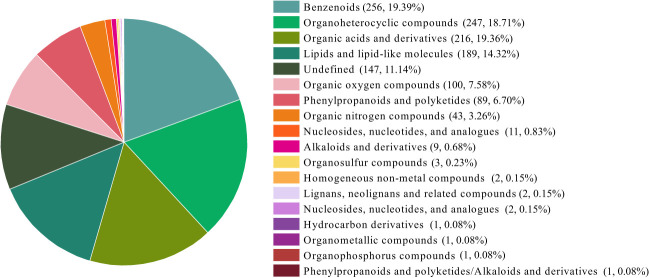
The classification of small molecules of AHA and SHA. The numbers in parentheses represent the amount of small molecule in each category and their proportion to the total number small molecules identified.

The 1320 small molecules classified into eighteen categories according to their chemical properties (**Figure 2**). The six categories that accounted for a higher percentage were benzenes, organic heterocyclic compounds, organic acids and derivatives, lipids and lipid-like molecules, organic oxygen compounds, phenylpropanoids and polyketides.

As PCA is an unsupervised data analysis method, orthogonal partial least squares discriminant analysis (OPLS-DA) was further employed to filter out the noise unrelated to the classification information and improve the parsing ability and effectiveness of the model. The OPLS-DA plot (**Figure S1**) of the two samples showed a clear separation. The OPLS-DA model was also used to obtain the variable weight values (VIP) and measure the intensity and explanatory power of the effect of each small-molecule substance on the classification discrimination of the AHA and SHA groups. Thereafter, multivariate analysis of OPLS-DA and univariate analysis of *t*-test were used to identify differential accumulated component ions. The use of VIP≥1 and *p ≤* 0.05 as cutoff thresholds revealed 357 differentially expressed positive ions, of which 234 and 123 shown FC (fold change, AHA/SHA) >1 and FC<1, respectively (**Support Material 1**, **Figure S2**).

Among the 510 small molecules with significant differences, 17 were attributed to hormones and their derivatives (**Table 1**). With exception of Trans-zeatin and Forchlorfenuron, two plant and hormone-like substances with an ionic strength FC<1, the remaining 15 bioactive substances showed FC>1, especially 5-methoxytryptophan, Gibberellin a4 and gamma.-hydroxybutyric acid, whose FC were 486.10, 263.50 and 109.57, respectively. Notably, we also found that AHA and SHA contain a variety of substances with bacteriostatic and anthelmintic effects, such as fludioxonil, vanillic acid, triadimefon, Penthiopyrad, dinex, dichlormid, etc (**Support Material 1**).

**Table 1:** Analysis of differentially abundant phytohormones and its derived small molecules.

Components	VIP	Fold Change	p-value	m/z	rt(s)
Gibberellin a4	2.04	263.50	7.92E-06	297.14446	351.356
Jasmonic acid	4.23	98.18	4.67E-05	193.09733	196.536
Dopamine	2.24	91.95	0.0017	137.05984	196.68
Melatonin	1.82	44.66	3.11E-07	255.11309	66.3665
Indolelactic acid	2.08	18.89	0.0005	206.08133	110.1555
(+)-.Alpha.-tocopherol	1.35	16.12	0.0027	165.09113	268.667
1 H-indole-3-carboxylic acid, 1-[2-(2,5-dimethylphenoxy)ethyl]	1.95	13.17	0.0025	292.11777	357.409
6-hydroxymelatonin	1.06	7.95	1.38E-10	232.09689	108.6255
5-methoxytryptophan	4.77	486.10	1.63E-11	233.08499	66.7377
N-phthalyl-l-tryptophan	1.08	52.47	0.0002	333.0663	239.1855
Indole-3-acetamide	1.19	15.78	0.0020	173.08133	255.88
Gamma-hydroxybutyric acid	1.41	109.57	1.16E-08	103.03909	332.3425
Aldosterone	1.01	12.50	0.0004	359.19024	239.082
3-bromo-5-phenylsalicylic acid	1.11	70.97	6.97E-08	246.95881	378.625
Trans-zeatin	2.21	0.19	9.63E-09	220.1121	60.8938
Forchlorfenuron	3.56	0.11	0.0027	248.07057	272.306

The VIP value is the value of variable weights obtained using the OPLS-DA model; Fold Change is the fold change of ionic strength of the substance in AHA and SHA; m/z is the mass-to-charge ratio of the substance; rt(s) is the retention time of the substance.

### 3.2 Phenotype changes in maize

Foliar applications of AHA and SHA exerted differing effects on maize growth. In plant treated with AHA, a greater number of expanded leaves, plant height, leaf area and leaf dry weight was observed (+4.72%, 6.12%, 12.87% and 13.29%, respectively) compared to CK treatment (*p*<0.05), but root growth was not modified (**Table 2**). Meanwhile, SHA treatment did not induce any significant changes in these parameters (except for the number of expanded leaves) compared to CK treatment. The increment in shoot leaf area after treatment with AHA was due to a small increment in total leaf number per shoot and a significant increment in average leaf size compared to SHA and CK treatments. In particular, compared with SHA and CK, the leaf area of AHA treatment group increased by 12.87% and 18.91%, respectively, and the leaf dry weight increased by 13.29% and 11.69%, respectively. Based on these results, AHA inducing more effective stimulation than the SHA.

**Table 2:** Results of phenotypic analyses of maize subjected to different treatments.

Treatment	Number of visible leaves	Number of expanded leaves	Plant height (cm)	Stem diameter (mm)	Leaf area (cm^2^)	Leaf dry weight (g per plant)	Stem dry weight (g ·plant^-1^)	Root dry weight (g ·plant^-1^)
AHA	15.00 ± 0.47	11.10 ± 0.32 a	187.33 ± 7.47a	18.47 ± 1.23	3143.70 ± 237.72a	15.77 ± 1.21 a	13.38 ± 2.70	6.83 ± 1.77
SHA	14.60 ± 0.52	11.00 ± 0 a	183.25 ± 3.5ab	17.78 ± 1.52	2785.16 ± 296.09b	13.92 ± 1.67 b	11.62 ± 2.54	5.36 ± 1.14
CK	14.60 ± 0.52	10.60 ± 0.52 b	176.53 ± 9.46b	18.32 ± 1.39	2643.73 ± 311.57b	14.12 ± 1.08 b	13.28 ± 1.97	6.95 ± 0.28

Values are presented as the means ± standard errors (n = 4). Different letters in the same group indicate statistically significant differences at *p* ≤ 0.05.

### 3.3 Nutrient absorption

**Figure 3:**
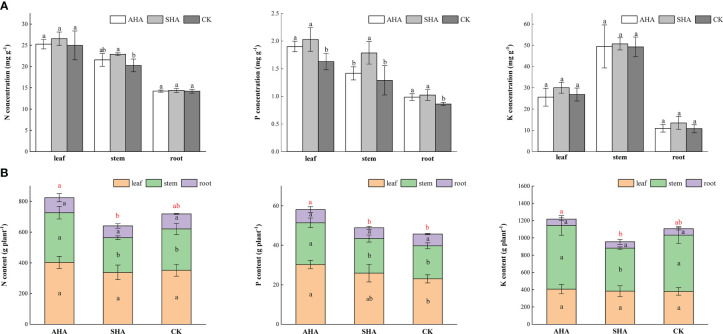
Different portions of maize subjected to various treatments differ in their nutrient concentration **(A)** and content **(B)**. Different letters indicate statistically significant differences at *p* ≤ 0.05. Different red letters indicate statistically significant differences of whole plant nutrient content at *p* ≤ 0.05. Vertical bars indicate means ± standard errors (n=4).

Both foliar spray treatments increased the P concentration in the leaves, stems and roots of maize plants (**Figure 3A**). The AHA and SHA treatments increased the stem N concentration by 6.4% and 13.04%, respectively, compared with the control treatment, but only the SHA group was declared to be significantly different from control plants (*p*<0.05). The K concentrations in maize were not affected by the tested biostimulants (**Figure 3A**). In contrast to stem N concentration, the N content in the plant following the AHA treatment increased by 43.52%, 20.62% compared with that observed after SHA and control treatments, respectively, due to the higher stem dry weight (**Figure 3B**). Similarly, the AHA treatment significantly increased the N, P and K content of the whole plant than that in SHA treatment plant (*p*<0.05, **Figure 3B**).

### 3.4 Photosynthetic parameters

**Figure 4:**
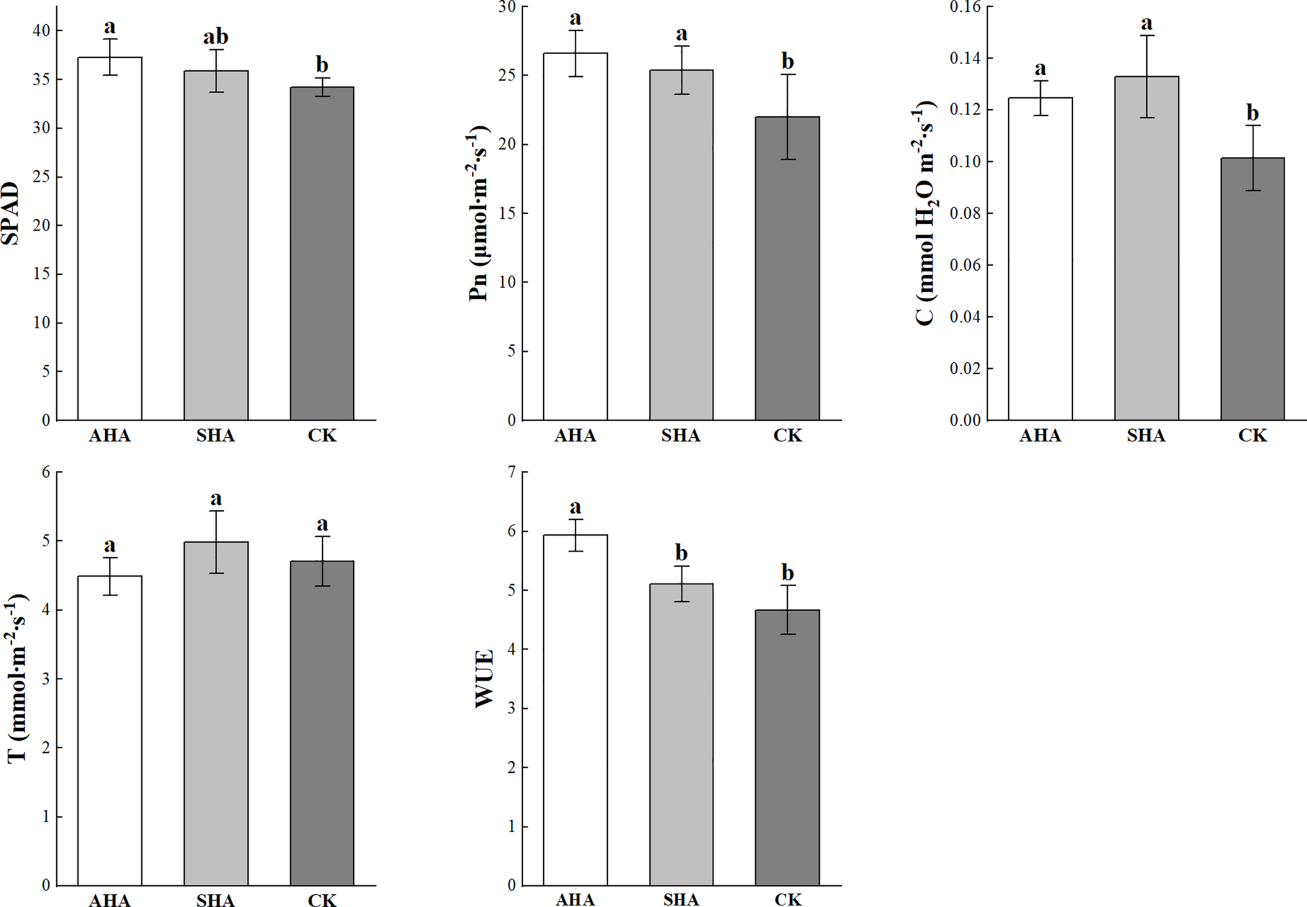
Effects of different treatments on photosynthesis. Different letters indicate statistically significant differences at *p* ≤ 0.05. Vertical bars indicate means ± standard errors (n=4). SPAD, relative chlorophyll content measured portable SPAD meter; Pn, net photosynthetic rate; C, stomal conductance; T, transpiration rate; WUE, instantaneous water use efficiency.

A positive effect of HSs was also been observed on the photosynthesis in maize leaves (**Figure 4**). Compared to the control treatment, plants subjected to the AHA foliar spraying treatment exhibited a significantly increased relative chlorophyll content (SPAD value) and C and subsequently displayed an increase in net photosynthesis; whereas there was a slight increase in SPAD values of SHA-treated leaves, their C and Pn were similarly significantly increased. There was no statistical difference in leaf SPAD, C and Pn between the humic acid treatments (**Figure 4**).

### 3.5 Carbon and nitrogen metabolism-related enzymes and substances

**Figure 5:**
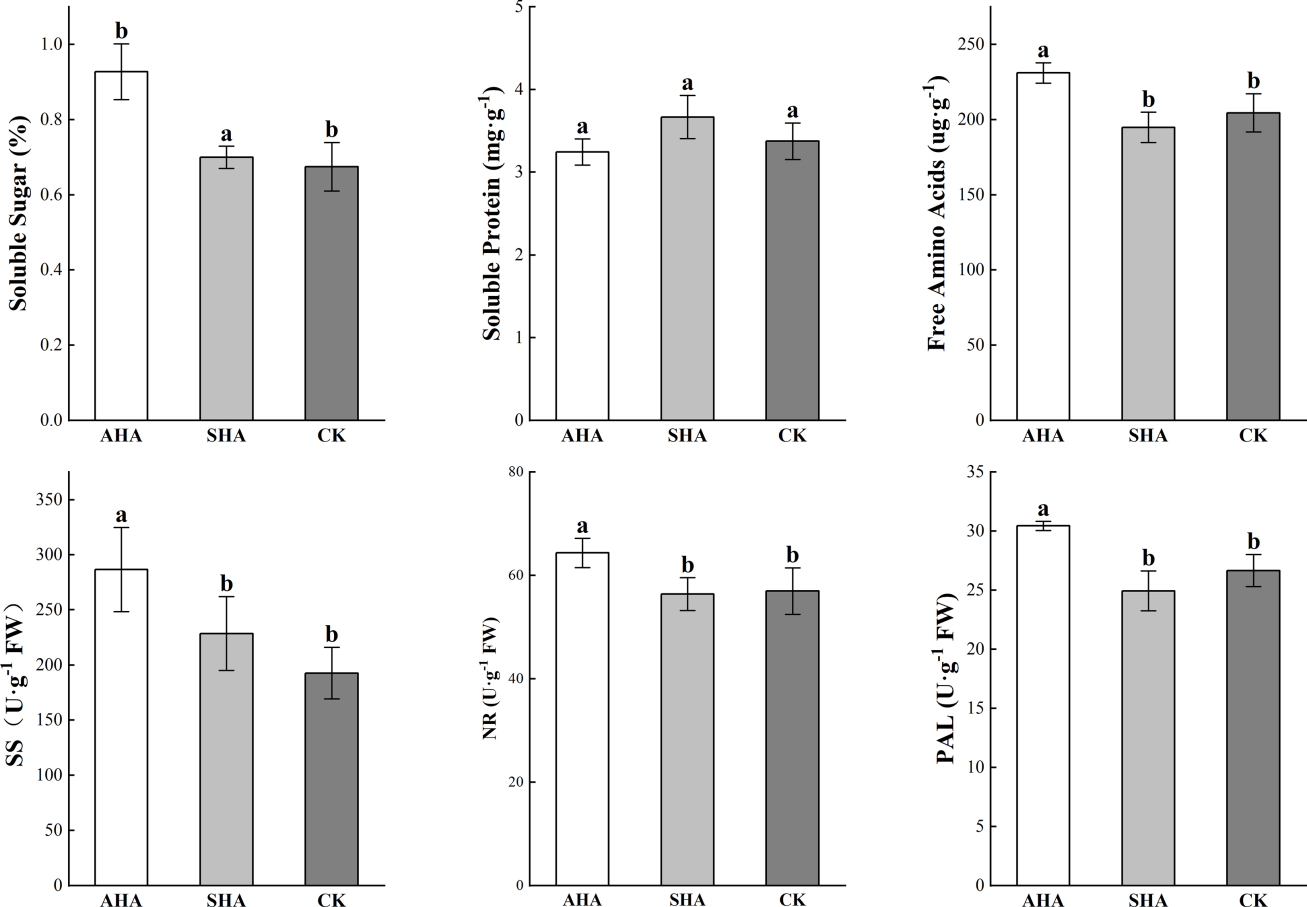
Carbon and nitrogen metabolites and their related enzyme activities in maize leaves subjected to different treatments. Different letters indicate statistically significant differences at *p* ≤ 0.05. Vertical bars indicate means ± standard errors (n=4). SS, sucrose synthase; NR, nitrate reductase; PAL, phenylalanine ammonia-lyase.

Compared to the control group, plants subjected to the AHA foliar spraying treatment presented higher levels of soluble carbohydrates and free amino acid content, but did not exhibit a change in the leaf protein concentration (**Figure 5**). SHA treatment did not exert any significant effect on leaf soluble carbohydrate concentrations but induced a slight reduction in free amino acid concentration when compared to CK treatment. SS, NR and PAL activities in maize leaves were significantly increased by AHA treatment than that in SHA and CK treatments plant (*p*≤0.05, **Figure 5**).

### 3.6 Metabolomic profile

#### 3.6.1 Effects of different treatments on overall metabolic profile

**Figure 6:**
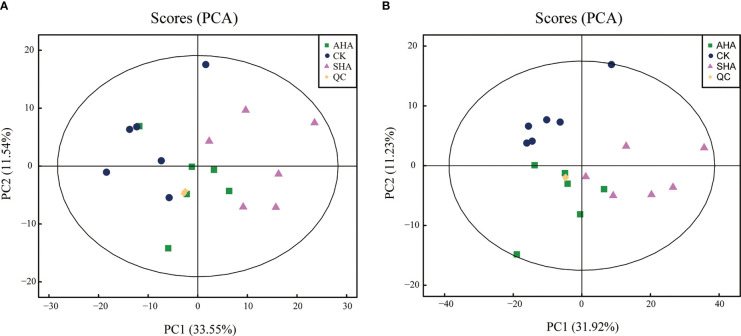
PCA score plots of metabolite profiles data obtained for maize leaf in in positive ions mode **(A)** and negative ions mode **(B)** (n=6).

**Figure 7:**
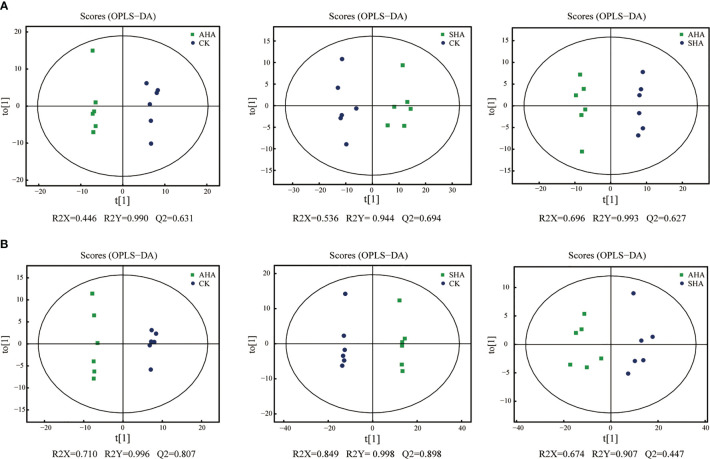
OPLS-DA score plots of maize leaf metabolome in positive ions mode **(A)** and negative ions mode **(B)**. The values at the bottom of the figure show the parameters evaluated in the model that were obtained after 7-fold cross-validation.

An untargeted metabolomics approach based on UPLC-QTOF-MS was used to obtain the metabolite profiles of maize leaves. Notably, 1373 characteristic peaks were detected and 1272 metabolites were identified. The PCA score plot (**Figure 6**) showed that the positive and negative ion modes explained 45.09% and 43.15% of the total variance, respectively. All groups in the positive ion mode were separated along PC2, and in the negative ion mode the treatment groups and CK were separated along PC2 and between treatment groups along PC1, reflecting the differences in their metabolic profiles. Metabolomics data were compared between the three treatment groups using OPLS-DA for a two-by-two comparison to maximize intragroup separation. The OPLS-DA scores for metabolomic profiles off the various treatment groups indicated a definite distinction between the groups (**Figure 7**).

#### 3.6.2 Identification of the discriminatory metabolites

**Figure 8:**
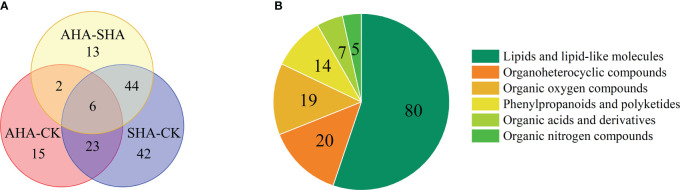
**(A)** Venn diagram of differentially abundant metabolites in maize leaves from different treatments groups. **(B)** the classification of differentially abundant metabolites in maize leaf.

Based on the VIP threshold (VIP>1) in OPLS-DA combined with *p*<0.05 from the univariate statistical analysis, 145 differentially abundant metabolites were screened. The identification of metabolites associated with the effects of humic acid spraying contributes to the understanding of the mechanism of action of HS. A Venn diagram (**Figure 8A**) was constructed to depict the differences and shared differentially abundant metabolites between the AHA treatment and CK groups, between the SHA treatment and CK groups, and between the AHA treatment and SHA treatment. Six shared differentially abundant metabolites were identified in all pairwise comparisons (trehalose, palatinose, diadinochrome A, fecosterol murrayone and 5-O-feruloylquinic acid). Compared to the CK treatment, there were 15 specific differentially abundant metabolites were observed in the AHA treatment group (including HDMBOA-Glc and Hirsuteine) and 42 specific differentially abundant metabolites were observed in the SHA treatment (such as jasmonic acid, linolenic acid and diosmin).

The 145 differentially abundant metabolites were classified into six categories according to their chemical properties (**Figure 8B**). Among which 80 metabolites (55.17% of the differentially abundant metabolites) were classified as lipids and lipid-like molecules, which are integral cellular components of the organism and play a key role in regulating normal cellular physiology and function, indicating that the different treatments significantly interfered with lipid metabolism in maize leaves. Phospholipids are a large class of lipids and lipid-like molecules and essential components of plant cell membranes and cellular signal transduction cascade reactions. In this study, 13 classes of phospholipid molecules and 53 species were identified. Eleven differentially abundant phospholipid molecules were screened in the comparison of SHA and CK treated maize leaves, of which 10 differentially abundant phospholipid molecules had an FC>1 (**Table S1**). Ten differentially abundant phospholipid molecules were screened in the comparison of AHA and SHA treated maize leaves, of which 9 differentially abundant phospholipid molecules had an FC>1 (**Table S1**). In summary, a generally significant increase in phospholipid composition was observed in SHA treated maize leaves, suggesting a disorder of lipid metabolism.

**Figure 9:**
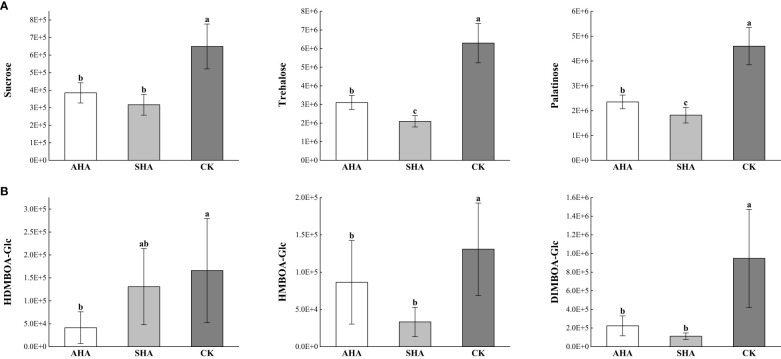
Relative abundance of differential metabolites in Maize leaf in different treatments. **(A)** relative abundance of carbohydrates; **(B)** relative abundance of Benzoxazinones (Bxs).

Nine of the nineteen organic oxygen compounds were carbohydrates and carbohydrate conjugates (**Figures 8B**, **9A**), which are the energy carriers and the building blocks of primary and secondary metabolism in plants and play an important role in plant growth. Therefore, changes in carbohydrate response suggest that carbon redistribution and mobilization are important features of maize leaves subjected to different treatments.

Among other metabolites, the response of benzoxazinone-glucoside (Bxs-Glc) differed among the different treatment (**Figure 9B**), and the relative abundance values of DIMBOA-Glc and HMBOA-Glc were significantly lower in the leaves of both groups subjected to humic acid spray treatments than in the CK treated leaves, while HDMBOA-Glc was a specific differentially abundant metabolite detected in the AHA treated leaves.

#### 3.6.3 Alterations in metabolic pathways in maize leaves in response to different treatments

Changes in the abundance of metabolites reflect the inhibition or activation of specific metabolic pathways, which also represent the regulation of metabolism. Annotation of the identified metabolites yielded 19 metabolic pathways, including starch and sucrose metabolism; the citrate cycle (TCA cycle); stilbenoid, diarylheptanoid and gingerol biosynthesis; ABC transporters; biosynthesis of unsaturated fatty acids; alpha-linolenic acid metabolism; tryptophan metabolism and carotenoid biosynthesis, among others (**Table S1**). The present study identified 21 differentially abundant metabolites produced by various metabolic pathways, including trehalose, sucrose, phenmetrazine, trimethoprim, meperidine, nandrolone, 2,5-Dihydroxybenzoic acid, 3-p-coumaroylquinic acid, stearic acid, trimethoprim, nostoxanthin, antheraxanthin, 9s,13r-12-oxophytodienoic acid, jasmonic acid, linolenic acid, safranal, cyromazine, cinobufagin, cholestenone, tryptophol, peonidin-3,5-O-di-beta-glucopyranoside, phenmetrazine, pergolide.

**Figure 10:**
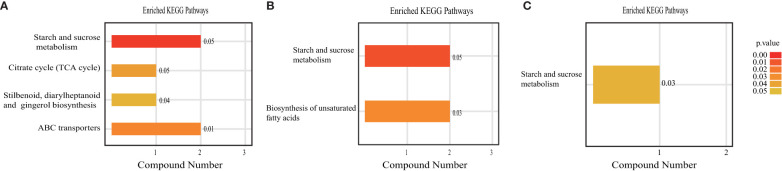
KEGG enrichment analyze of differentially expressed metabolites in maize leaf. The values after bars indicate enrichment factor. **(A)** AHA vs CK, **(B)** SHA vs CK, **(C)** AHA vs SHA.

An enrichment analysis of metabolic pathways was subsequently performed to identify significantly affected metabolic and signal transduction pathways (**Figure 10**). Compared to the CK treatment, AHA treatment resulted in the reorganization of four metabolic pathways: starch and sucrose metabolism, TCA cycle, stilbenes, diarylheptanes, and curcumin biosynthesis, and ABC transport (**Figure 10A**). Of these, starch and sucrose metabolism, the TCA cycle is central to carbohydrate metabolism. Two metabolic pathways-starch and sucrose metabolism and unsaturated fatty acid biosynthesis-were modified by SHA therapy (**Figure 10B**). Only one metabolic pathway, starch and sucrose metabolism, was enriched when comparing the metabolic enrichment status of leaves treated with AHA and SHA (**Figure 10C**). One biological pathway, starch and sucrose metabolism, was altered in the comparisons of all treatment groups when they were compared, indicating that this pathway is sensitive in leaf tissue.

**Figure 11:**
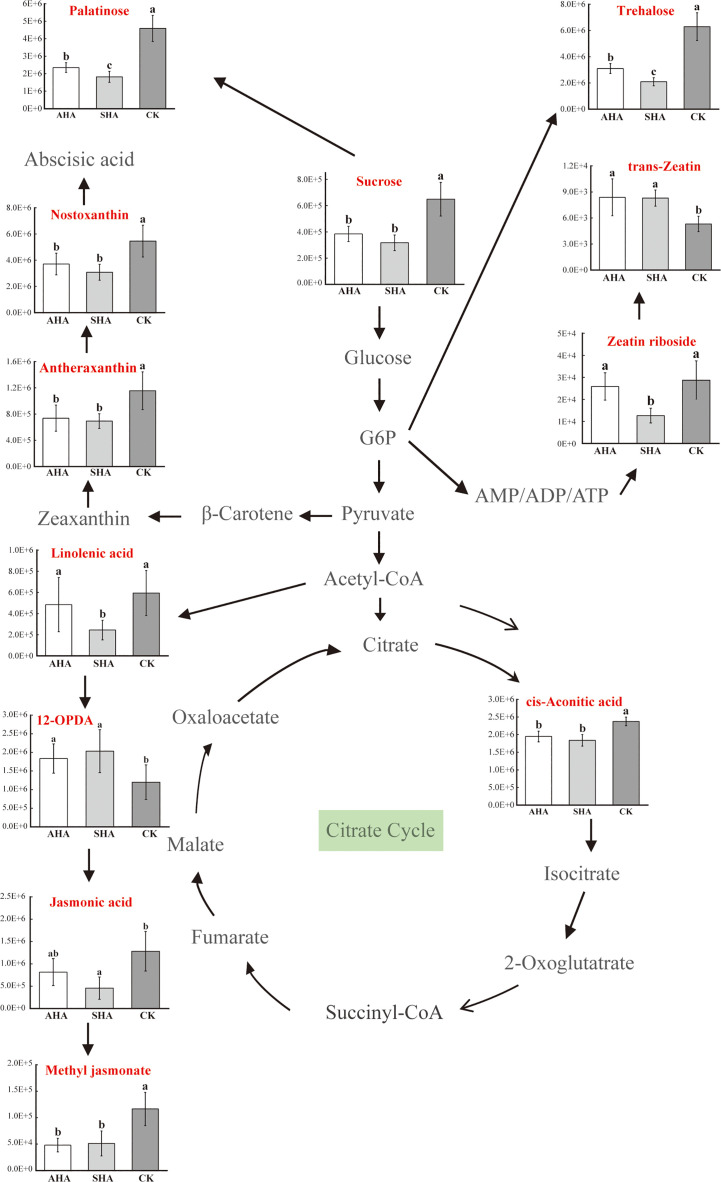
Simplified metabolic pathways of maize leaves exposed to different treatments. G6P, glucose-6-phosphate; AMP/ADP/ATP, adenosine monophosphate/adenosine diphosphate/adenosine triphosphate.

However, considering the effects of interconnected networks of metabolic pathways leads to a more thorough understanding. A more detailed sketch map of the metabolite network modifications in the main metabolic biosynthetic pathway which includes glycolysis, the TCA cycle, abscisic acid biosynthesis, and jasmonic acid biosynthesis, was shown in **Figure 11**. Regarding phytohormones, nostoxanthin and antheraxanthin, which are intermediates in the abscisic acid biosynthesis pathway, were down-regulated by both humic acid spray treatments when compared to CK treatment (**Figure 11**; **Table S1**), which may have led to a reduction in abscisic acid synthesis. Compared to the CK treatment, the accumulation of linolenic acid, jasmonic acid, and methyl jasmonate was decreased by SHA treatment in terms of the biosynthesis of jasmonic acid; AHA treatment only reduced the accumulation of methyl jasmonate, it had no effect on the accumulation of α-linolenic acid, 12-OPDA, or jasmonic acid (**Figure 11**; **Table S1**). Additionally, different treatments exerted distinct effects on the cytokinin production pathway. Compared to the CK treatment, SHA treatment increased trans-zeatin accumulation (VIP=0.18, *p*<0.05), while AHA treatment increased trans-zeatin accumulation (VIP=0.20, *p*<0.05), but substantially decreased zeatin nucleoside accumulation (VIP=0.44, *p*<0.05).

## 4 Discussion

HSs increase plant growth, root system development (Canellas et al., 2002; Zandonadi et al., 2007; Trevisan et al., 2010), and several physiological processes (Mora et al., 2010; Conselvan et al., 2017). HSs may be applied directly to the soil or sprayed over leaves. Both applications are commonly utilized in agricultural practices, the majority of the documented effects of HSs on plant growth are derived from studies of short-term HSs application to roots in the greenhouse, whether in hydroponics or soil. However, other studies have highlighted the ability of HSs to promote plant growth when applied as a foliar spray. The effects of root-applied HSs are typically distinguished by two different mechanisms: one is indirect and involves enhancing the chemical, physical, and biological qualities of soil; the other is a direct result of the effects of the active ingredients on the controlling of growth processes, nutrient transport mechanisms, and primary and secondary metabolism. HSs applied to the leaves have little impact on the soil or rhizosphere, but there are major responses and interactions that take place between the soil and the roots that boost nutrient absorption. In this case, the mode of action of HSs seems to involve unique plant nutritional, metabolic and physiological responses.

De Hita et al. (2020) designed experiments to distinguish the mechanisms of action of foliar treatment of sedimentary humic acid on plant development compared with root application. Both application strategies promoted the growth of aboveground parts and roots. The potential of sedimentary humic acid to increase cytokinin concentration in aboveground tissues and indole-3-acetic acid concentrations in roots was shared by its foliar and root applications. Although foliar-applied sedimentary humic acid significantly increased shoot and root growth, it had no effect on the levels of nutrients in the leaves. Kishor et al. (2021) and Hernandez et al. (2014) found that foliar application of humic acid improved significantly the plant growth, yield and physiological processes in coffee and lettuce. The results presented here reveal various effects of two HSs from distinct origins on plant growth and highlight the challenge of research involving complex mixtures of poorly characterized components. Previous studies have observed the full range of plant responses to HSs, ranging from an inhibitory effect to a stimulatory effect (Rose et al., 2014; Canellas et al., 2015). In this study, foliar-applied AHA produced a larger biomass, plant height, leaf area, relative chlorophyll content and net photosynthesis, all of which indicated rapid resource utilization and subsequent rapid growth. However, AHA treatment did not cause any change in plant nutrient concentrations (except for the leaf P concentration, **Figure 3**). In combination with the findings of De Hita et al. (2020), this result may be attributed to the lack of an increase in H^+^-ATPase activity in the root plasma membrane following foliar spraying. The diluting effect may also account for the comparability of N and K concentrations in the AHA-treated plants to the control plants due to the relatively large plant size (**Figure 3**; **Table 2**). Furthermore, the AHA treatment significantly increased leaf dry matter while having no effect on plant root growth (**Table 2**). A possible explanation for this phenomenon is that AHA treatment reduces root redundancy in maize plants, which is highly energy intensive and has significant constraints on the formation of economic yield. It has long been accepted wisdom that the larger the root system, the more water and nutrients the plant obtains, and the greater the output. However, excessive root growth will unavoidably result in redundancy, which will have an effect on aboveground growth (Hu et al., 2011). In addition, the application of AHA to maize leaves significantly enhanced photosynthesis, which in turn increased the amount of soluble sugar in the leaves. Meanwhile, AHA treatment also raised sucrose synthase activity, therefore, promoting the conversion and utilization of sugars in leaves, leading to an increase in the aboveground biomass.

Despite the considerable amount of data concerning the physiological and biochemical effects of these compounds on specific metabolic pathways, little information is available on the global effects exerted by HS on metabolism. As stated above, HSs appears to influence plant physiology through a cascade mechanism targeting multiple metabolic steps. Their hormone-like activity may have a role in this process, but other unidentified signaling networks may also contribute. A comprehensive study of the plant response to these substances might significantly contribute to improving our understanding of the molecular mode of action of these substances. Thus, we employed metabolomics to perform a large-scale investigation into the overall metabolic effects of HSs on maize. In this study, an untargeted metabolomics approach based on UPLC-QTOF-MS was used to obtain metabolite profiles of maize leaves, and PCA revealed differences in the metabolic profiles between the two humic acid treatment groups and CK group, as well as between the two humic acid treatment groups, which verified the different effects of these two humic acids on maize growth. Integration of KEGG enrichment analysis of differential metabolites revealed that compared to the CK group, AHA treatment reshaped four metabolic pathways: starch and sucrose metabolism, the TCA cycle, stilbenes, diarylheptanes and curcumin biosynthesis and ABC transport, of which starch and sucrose metabolism and the TCA cycle are the core of carbohydrate and energy metabolism.

Sugar is an energy source for plants and can act as a signaling molecule during plant growth. Previous studies have shown that changes in the amount of free sugars in maize modulate the expression of growth regulatory genes (Gibson, 2005). In this study, the relative sucrose content in maize leaves treated with CK was higher than that in AHA treatment group, and the stomatal conductance of leaves was also observed to be lower than that in AHA treatment group, resulting in a significant reduction in leaf photosynthesis. Several research papers have documented an effect of HSs on carbohydrate metabolism, modifying the level and distribution of sugars in treated plants. As an illustration, Conselvan et al. (2018) suggested that the lower sucrose concentration in HS-treated *Arabidopsis* roots and leaves may be responsible for higher glycolytic activity to support HS-stimulated metabolic activities and hence boost plant growth. This result is supported by the discovery of increased sucrose synthase activity in the AHA-treated leaves in the current study (**Figure 5**). A logical assumption is that rapidly growing plants have an increased demand for source leaf (fully expanded leaves) photoassimilates after spraying with AHA. Therefore, the sucrose export rate from the source leaf should be higher, with less sucrose accumulated during the daytime. As anticipated, the sucrose concentration was lower than that in parallel control leaves, but the photosynthesis and soluble sugar contents of the AHA-treated source leaves were higher than those of parallel control leaves (**Figures 4**, **5**). Furthermore, excess sucrose may act as a signaling molecule to induce stomatal closure and reduce CO_2_ uptake in guard cell. The surplus sucrose is delivered toward the stomata by the transpiration stream and triggers stomatal closure via hexokinase when the rate of sucrose production exceeds the rate at which sucrose is loaded into the phloem (Kelly et al., 2013). Regarding trehalose, which is a typical stress metabolite, the larger amount of trehalose in CK treatment leaves may suggest the onset of a state of stress in plants. Indeed, plants typically contain little trehalose, as environmental stress increases, trehalose production starts to increase (Iordachescu and Imai, 2008; Wang et al., 2020).

Membrane lipids are also involved in the HS-induced modulation of plant signaling molecules. Phospholipids, which are plasma membrane component, are crucial for cell signaling, membrane trafficking, and apoptosis (Xue et al., 2009). The HS-based biostimulant treatment changed the phospholipids profile. The SHA treatment altered the levels of 11 foliar metabolites compared to CK, and 10 foliar metabolites compared to the AHA treatment. Therefore, maize leaves exposed to SHA may exhibit abnormalities in lipid metabolism, which exert a negative effect on the fluidity and integrity of cell membranes, thereby inhibiting maize growth and development.

Furthermore, we particularly noted that the content of benzoxazinones (BXs, such as BIMBOA-Glc, HDMBOA-Glc and HMBOA-Glc) was significantly lower in the leaves of maize sprayed with HSs than that in CK treatment. Benzoxazinoids (BXs) are regarded as a class of secondary metabolites involved in general defense in gramineae. Generally, BXs exist stably in plants in the form of glucosides, which are degraded into the corresponding glycosides and sugars to play a defensive role when the plant is attacked (Du Fall and Solomon, 2011). A general threshold for the induction of BXs production exists in plants upon pest and disease infestation and insect feeding, indicating that the plant mobilizes BXs for defense only after a certain level of damage by pathogens and insects occurs, which reduces the metabolic burden (Gianoli and Niemeyer, 1997). Furthermore, fulvic acid fractions have been shown to be effective in controlling several plant diseases, which were characterized by their capacity to induce host resistance towards a wide range of diseases or by directly acting as an antibacterial agent (Abdel-Monaim et al., 2011; Kamel et al., 2014). This also explained that the relative abundance of HDMBOA-Glc, HMBOA-Glc and DIMBOA-Glc in maize leaves sprayed with AHA and SHA in this study was generally lower than that in CK group, which may imply that maize leaves in the CK treatment group may consume more energy for defense.

The modulation of the phytohormone network contributed to the responses of plants to HS spray treatment. Nostoxanthin and antheraxanthin are two important intermediates in the synthetic pathway of the phytohormone abscisic acid (ABA). ABA is generally considered as the main phytohormone for plant resistance to abiotic stress, and ABA levels in plants increase when they are exposed to drought, salt, low temperature, high temperature, and injury. Therefore, ABA is also known as the adversity hormone (Klingler et al., 2010). Both HSs spray treatments significantly decreased the accumulation of these two metabolites, which may inhibit ABA synthesis. In addition, in this experiment, we observed that the stomatal conductance of CK-treated maize leaves was significantly lower than that of the humic acid spray treatment (**Figure 4**), which may also imply that the ABA content of humic acid-treated maize leaves was lower than that of the CK treatment, considering the relationship between stomatal conductance and ABA (Wang et al., 1994).

In the present study, SHA treatment decreased the accumulation of linolenic acid, a precursor in the jasmonic acid biosynthetic pathway, and the accumulation of jasmonic acid (JA) and methyl jasmonate (MeJA). Similarly, AHA treatment also decreased linolenic acid, JA and MeJA levels, but only the accumulation of MeJA was significantly different (**Figure 10**; **Table S1**). Since plant defense is an energy-intensive operation, JA is a stress hormone that controls how plants adapt to biotic and abiotic stress, forcing plants to stop active development (Ghorbel et al., 2021). In respect to cytokinins (CTK), both HSs treated maize leaves exhibited different levels of accumulation of tran-zeatin (tZ), but SHA treatment significantly decreased the accumulation of zeatin riboside when compared to CK treatment (**Figure 11**). The decreased accumulation of zeatin riboside, the main transport form of CTK, indicated that the CTK reserve in leaves treated with SHA was lower than that in leaves treated with CK and AHA (**Figure 11**). CTK negatively regulates the expression of chlorophyll-degrading enzyme-related genes, and induces the degradation of chlorophyll-degrading enzymes, resulting in an increase in the chlorophyll content, maintaining the normal photosynthetic capacity of leaves and the structure and function of the photosynthetic system, and extending the valid period of photosynthesis and the supply of photosynthetic compounds, it is the only known plant hormone that can delay leaf senescence (Talla et al., 2016). In the present study, the order of SPAD of maize leaves was AHA>SHA>CK, and the order of net photosynthesis was AHA, SHA>CK (**Figure 4**), consistent with the trend for tZ accumulation in leaves. In combination with the above discussion, the application of humic acid AHA increased the accumulation of tZ and down-regulated the relative levels of JA, MeJA and BXs in maize compared to CK, suggesting that there is a trade-off between growth and defense in plants sprayed with AHA and that this trade-off results in stimulation of plant growth.

Overall, although attributing the stimulation of the plant to few/some specific compounds is difficult, we speculate that the altered balance of phytohormones may have played a pivotal role in plant growth. Indeed, plant growth and development have consistently been shown to be tightly connected to hormone profile. Comparatively, much less is understood about the signaling pathways related to membrane lipids and the elicitation of secondary metabolism by HSs in terms of biotic stress induced systemic response to data, and further studies on this topic are advised.

For many years, soil scientists have endeavored to define the chemical characteristics and molecular structure of HSs and clarify how they can affect the growth and development of plants. Previous studies have observed that the complexity, chemical structure characteristics and biological activity of HSs determine their roles as plant growth biostimulators. Nardi et al. (2007) reported that low molecular weight HSs with high content of hydrophilic compounds significantly increased nitrogen uptake, the glycolytic pathway and the Krebs cycle in maize seedlings. The experimental results reported by Conselvan et al. (2018) revealed different response between plants treated with IAA and HS, emphasizing that responses evidenced by changes carbohydrate and amino acid concentrations were only partially attributed to the effects of IAA on HS substrates. The presence of other molecules present in the HS matrix can be used to determine their biostimulatory effect.

Most commercial products currently available for agronomic purposes are obtained from nonrenewable coal substrates such as peat, weathered coal coals and lignite, which raises the question of whether key structural aspects related to plant stimulation are also present in coal-related HSs. The opposite consequences of coal-derived HS on crop productivity (i.e., promoted or diminished effects) may be reinterpreted in light of the different structures of these materials. In the present study, the differences in plant growth and nutrient content between the AHA and SHA treatment groups (**Table 2**; **Figure 3**) suggested that the response of maize plant to HSs depends on its molecular composition. The positive effects of AHA on maize growth, even under well-watered and fertilized conditions, suggest a direct effect of compounds present in AHA on the plant. These two HSs have different elemental and molecular structural compositions (**Table 2**; **Figure 1**; **Support Material 1**). Combining the molecular structural features of the two HSs, we deduced that growth-promoting effect observed following AHA application was mainly attributed to GA4, JA, dopamine, and cross talk with the other hormones present at lower concentrations. Gibberellin plays an important role in regulating the growth and size of various organs during the period of plant nutritional growth, including hypocotyl growth, leaf extension, plant height regulation and root development. Its regulation of plant nutritional organ growth and development is mainly reflected in the promotion of cell elongation and promotion of cell division.

3,5-dichlorosalicylic acid is a chemical inducer of systemic acquired resistance of plants to a wide range of pathogens (Basson and Dubery, 2007) with an FC value of 404.91 in both HS-treated groups (**Support Material 1**). In addition, we found that the two HSs contained different levels of small molecules with fungicidal and insecticidal properties, including methamidophos, dichlormid, drazoxolon, triadimefon, penthiopyrad, pyrimethanil, perillaldehyde isocarbamid, mepanipyrim, moxamide, diethofencarb, triflumuron, bixafen, flusilazole, omethoate, spirodiclofen, fludioxonil, vanillic acid chlorogenic acid, quinolin-2-ol. These molecules may partly be responsible for the decreased activity of pathways regulating the biosynthesis of JA and ABA were in the HS-sprayed maize plants.

## 5 Conclusions

In this study, the molecular structures of two humic acids were characterized by ESI-UPLC-MS technique, and it was discovered that the molecular compositions of different sources of humic acids were similar with different relative abundances, and a total of 510 small molecules with significant differences were screened by combining multivariate statistical analysis. Pot spraying trials showed different effects of the two humic acids on maize growth and development: higher biomass, plant height, leaf area, chlorophyll content, and net photosynthesis were all observed in maize plants treated with AHA; the SHA treatment did not induce any significant changes in maize growth. Metabolomics revealed that a total of 145 metabolites were significantly altered between the different spray treatments. Further analysis revealed that leaf lipid metabolism, starch and sucrose metabolism and some phytohormone-related biological pathways were regulated by the humic acid spray treatments. Among them, there was a generally significant increase in phospholipid composition of SHA treated maize leaves compared to CK and AHA treatments. Regarding phytohormones, nostoxanthin and antheraxanthin, which are intermediates in the abscisic acid biosynthesis pathway, were down-regulated by both spray treatments. In addition, both humic acid treatments stimulated the accumulation of tZ, SHA treatment significantly down-regulated the accumulation of zeatin nucleosides when compared to CK treatment.

## Data availability statement

The original contributions presented in the study are publicly available. This data can be found here: MetaboLights, accession MTBLS7849.

## Author contributions

YW, YB designed the research. YW, GS, MX and CN performed the research. YW wrote the paper. YW, YB, BL, YL, LW and LN edited the paper. All authors contributed to the article and approved the submitted version.
