# Supplementary material for: Analysis of the molecular composition of humic substances and their effects on physiological metabolism in maize based on untargeted metabolomics
Source: Front Plant Sci. 2023 May 22;14:1122621. doi: 10.3389/fpls.2023.1122621 (PMC10239833; doi:10.3389/fpls.2023.1122621)
Supplement: Supplementary file 1 [file DataSheet_1.docx]

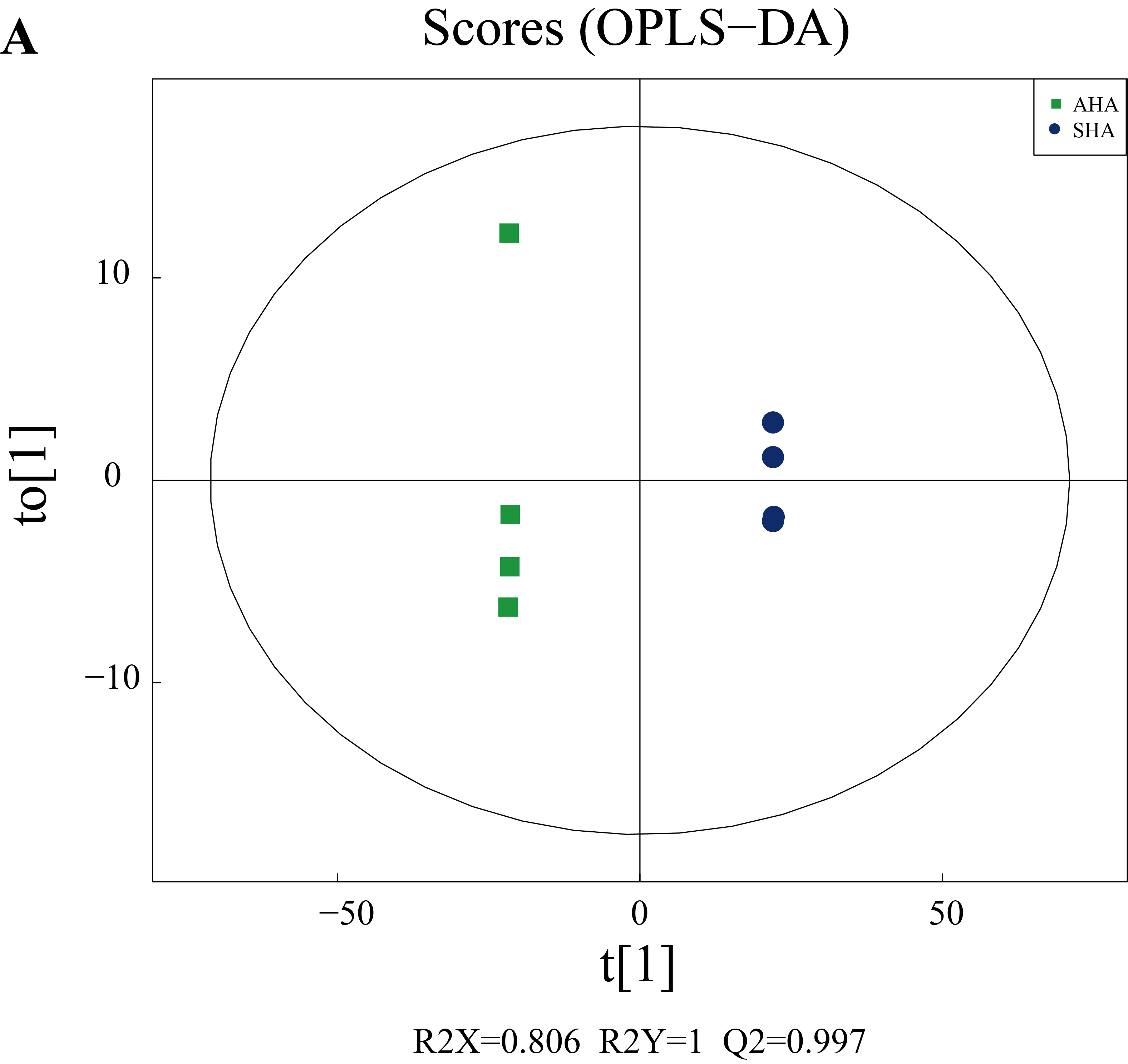

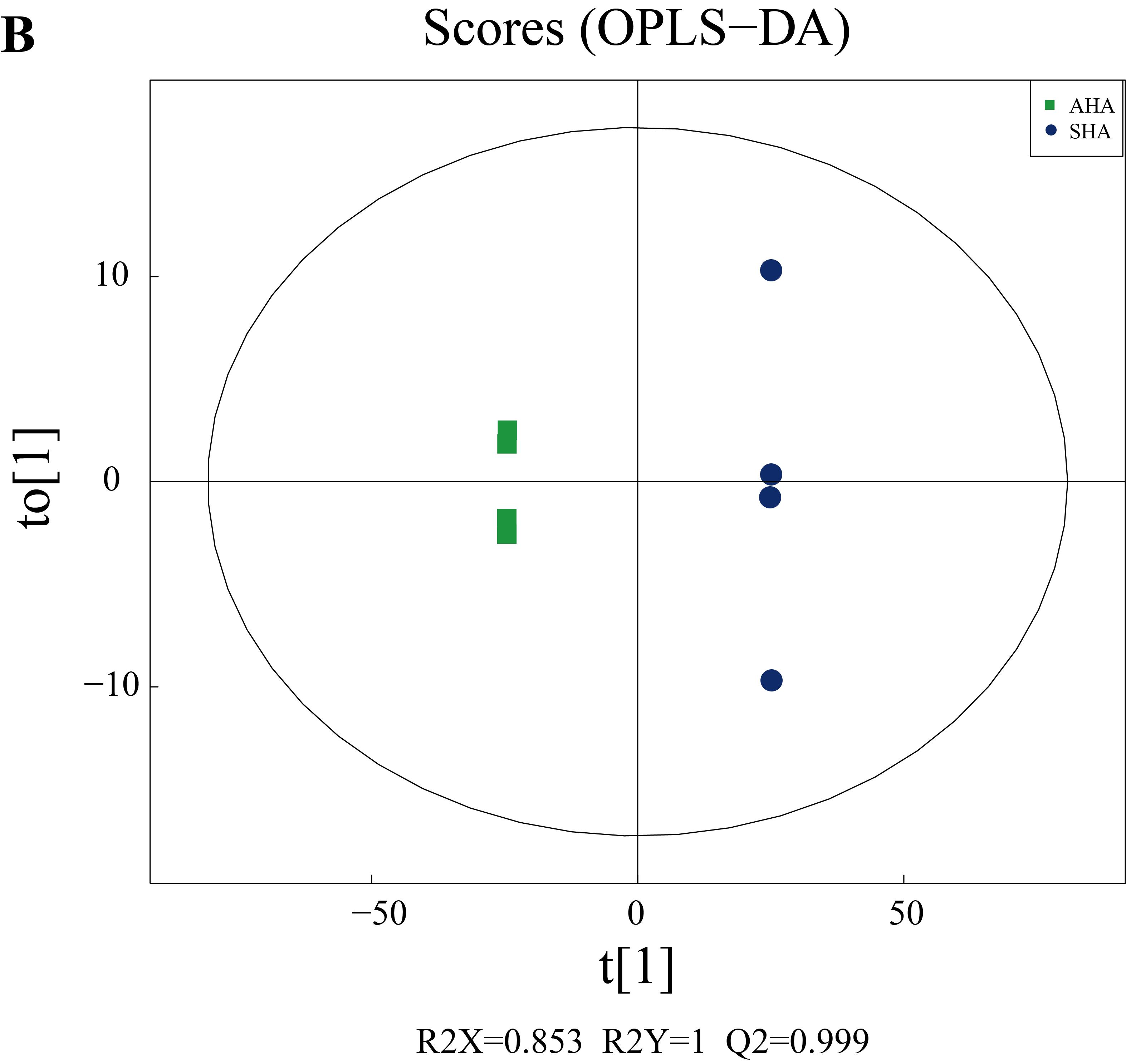


Fig. S1 OPLS-DA score plots of the differential metabolites in positive ions mode (A) and negative ions mode (B) of AHA and SHA.


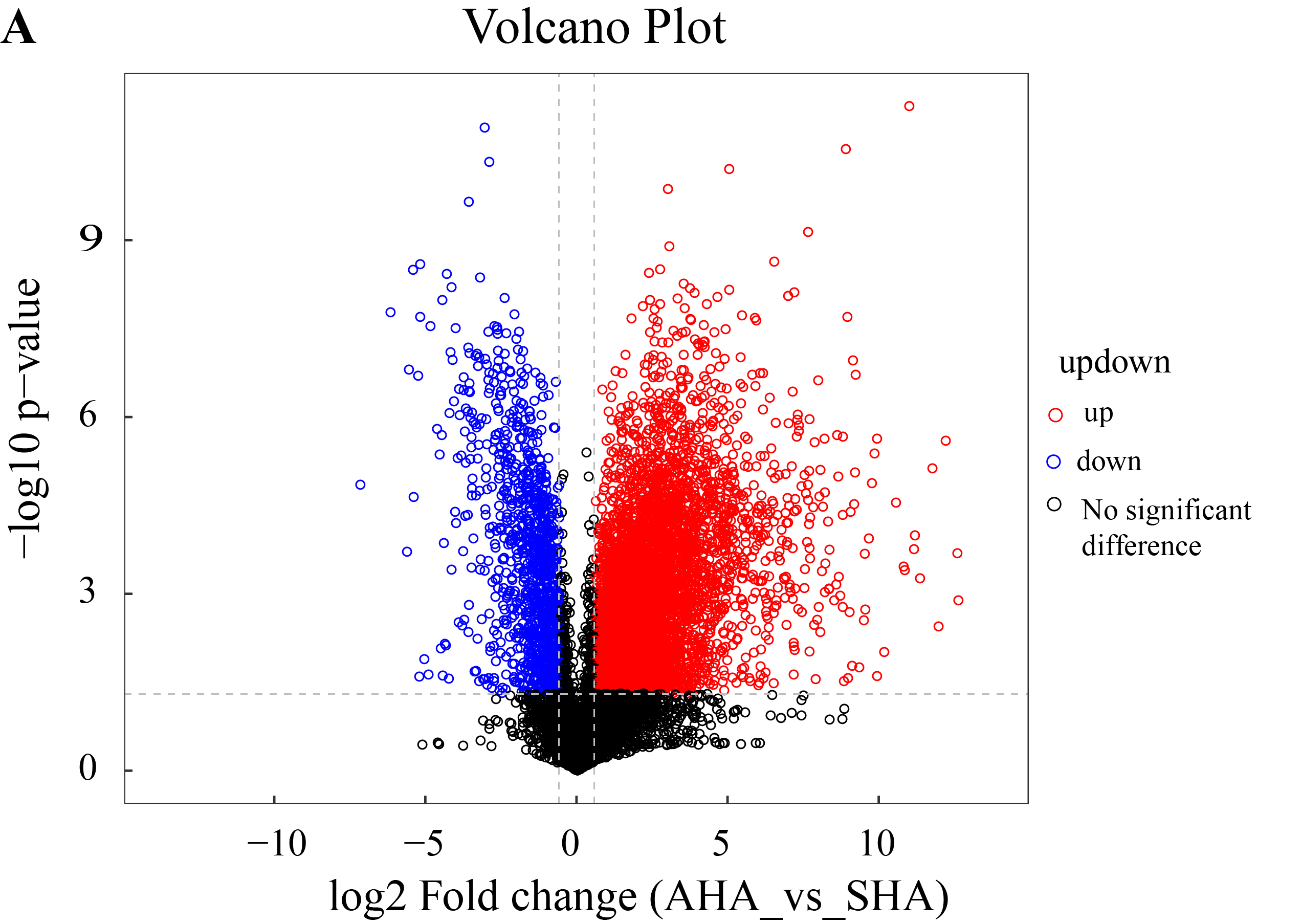

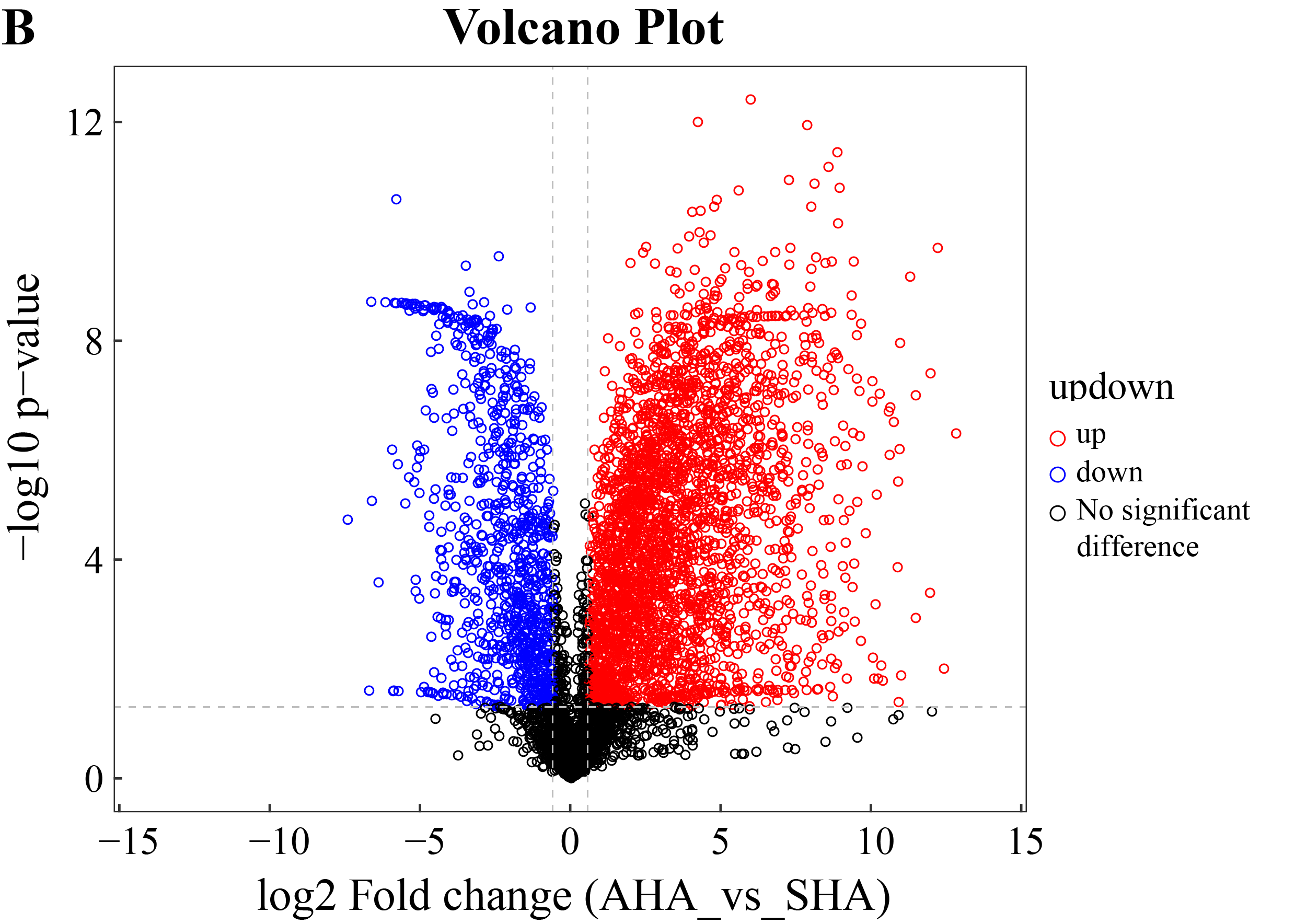

Fig. S2 Volcano-plot of the differential metabolites in positive ions mode (A) and negative ions mode (B) of AHA and SHA. The differential metabolites that up-regulated and down-regulated in abundance were indicated in red and blue, respectively.




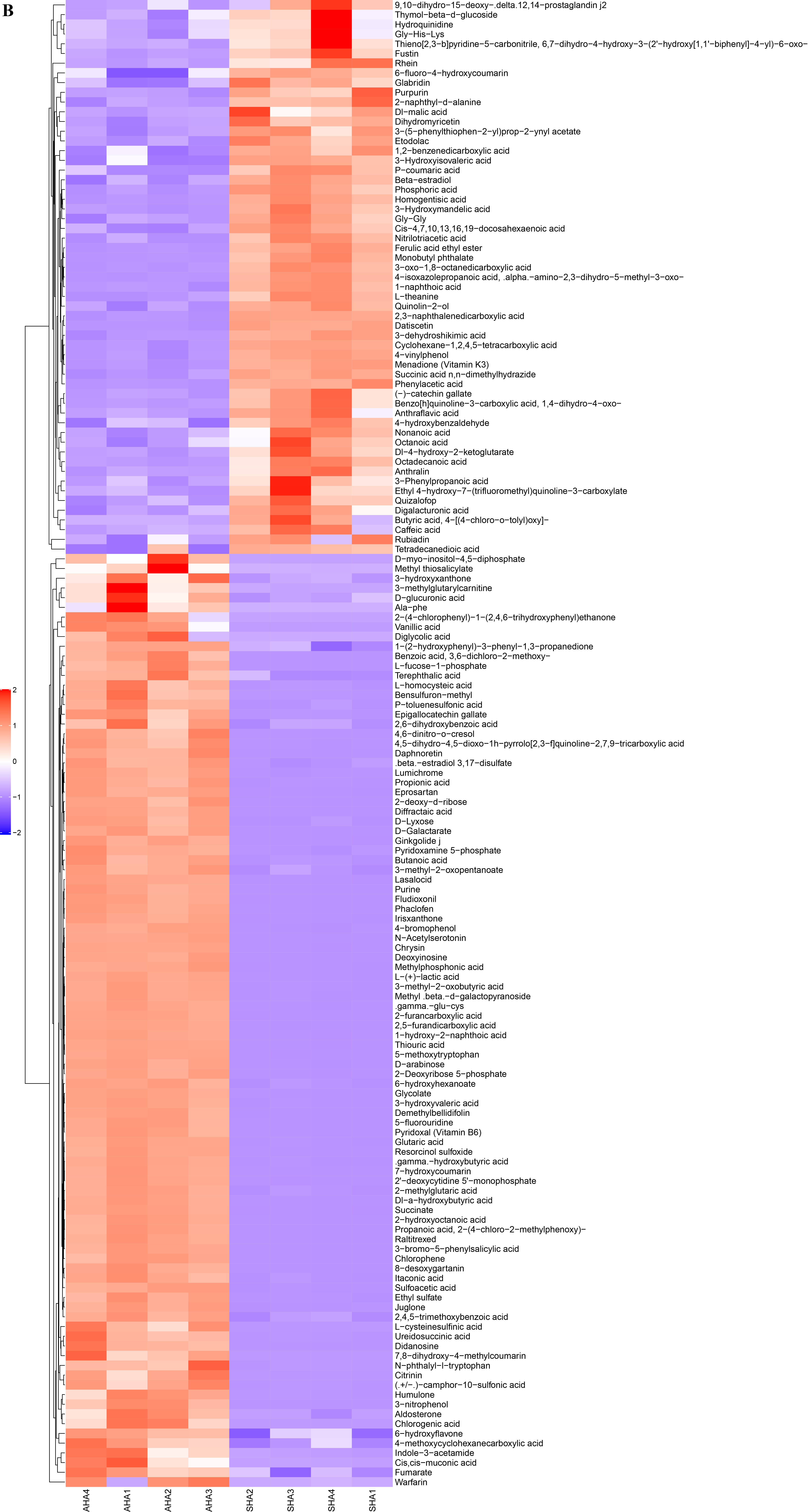


Fig. S3 Heat map and hierarchical cluster analysis of the differential components in in positive ions mode (A) and negative ions mode (B) (AHA vs SHA).


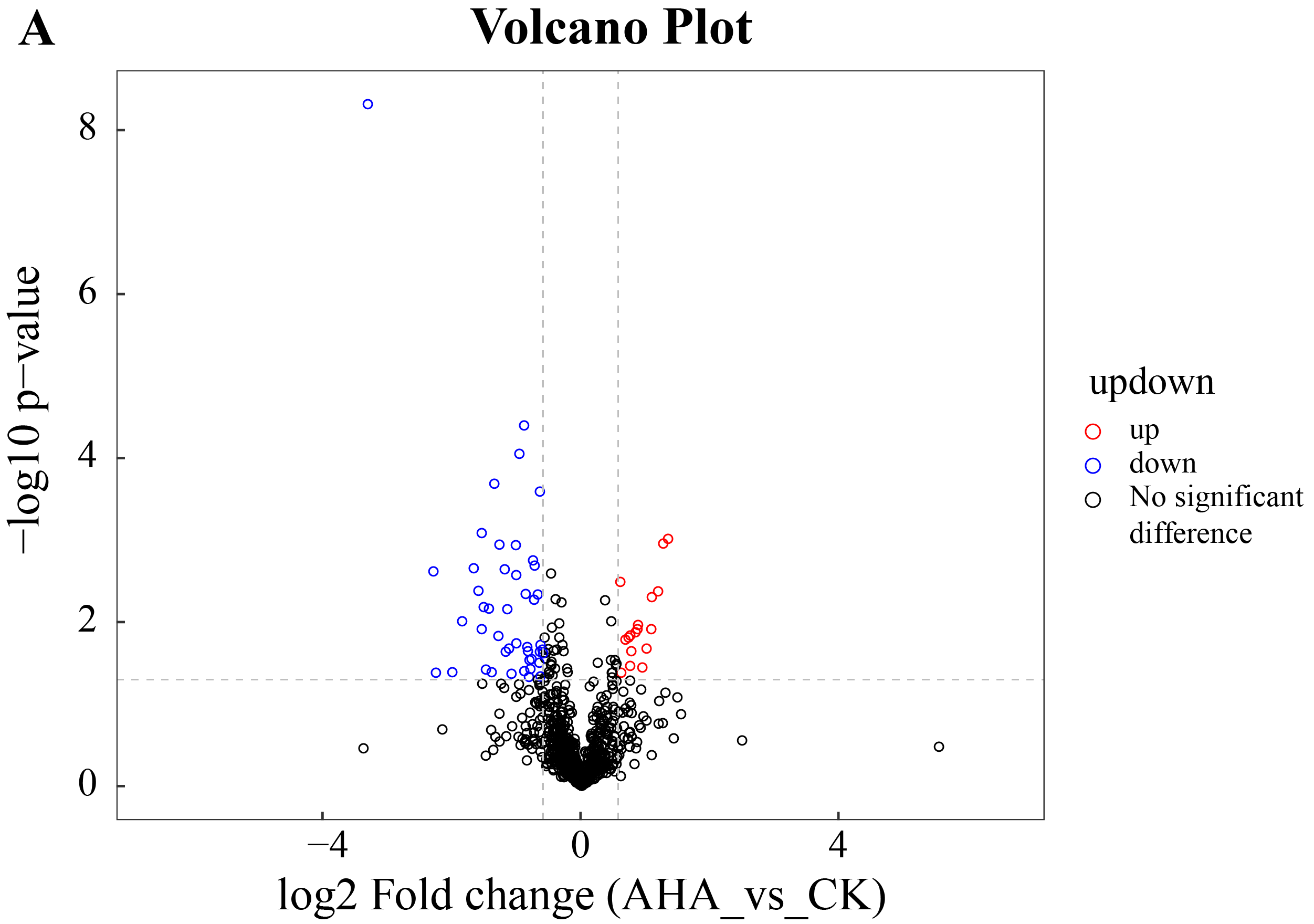

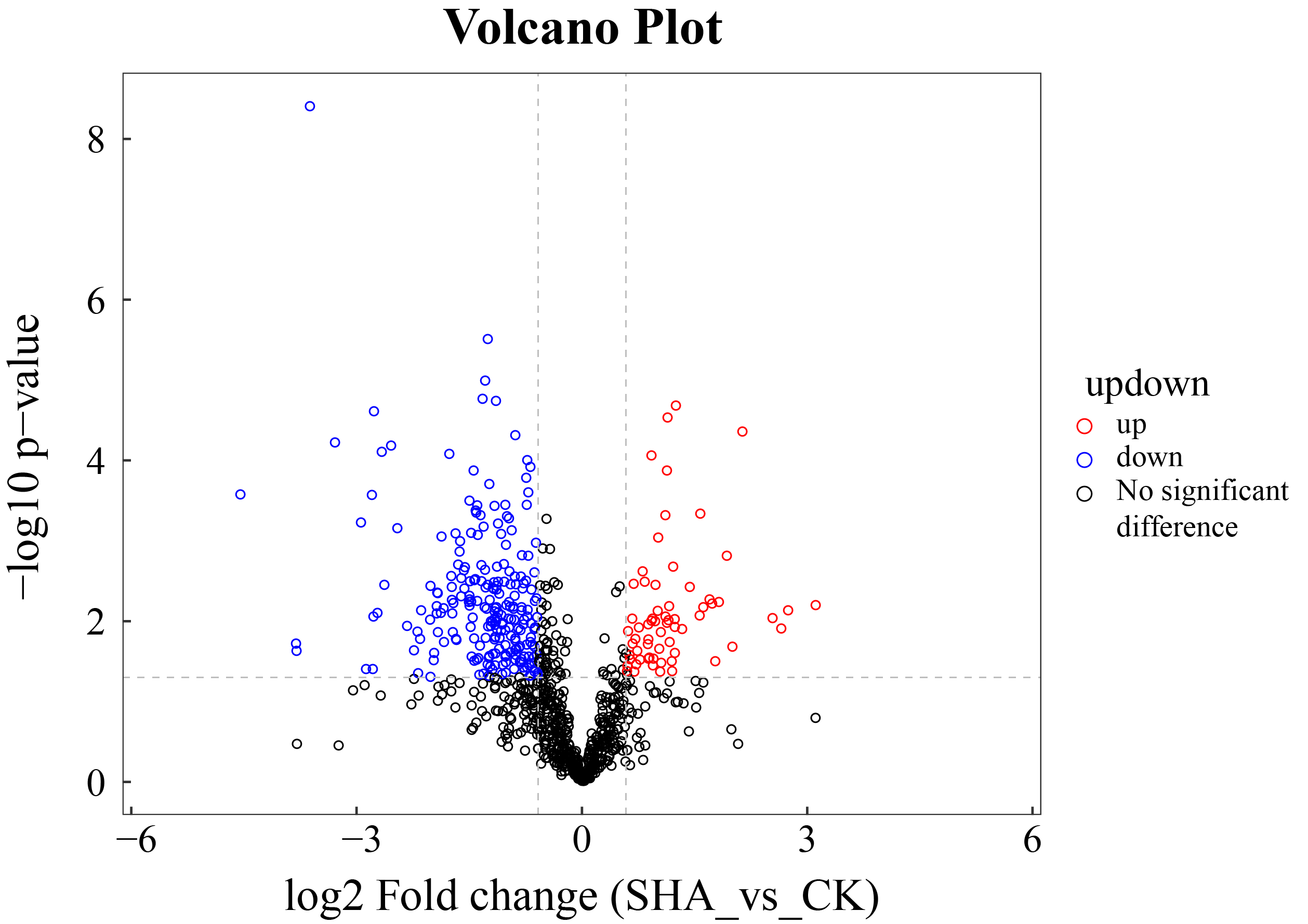

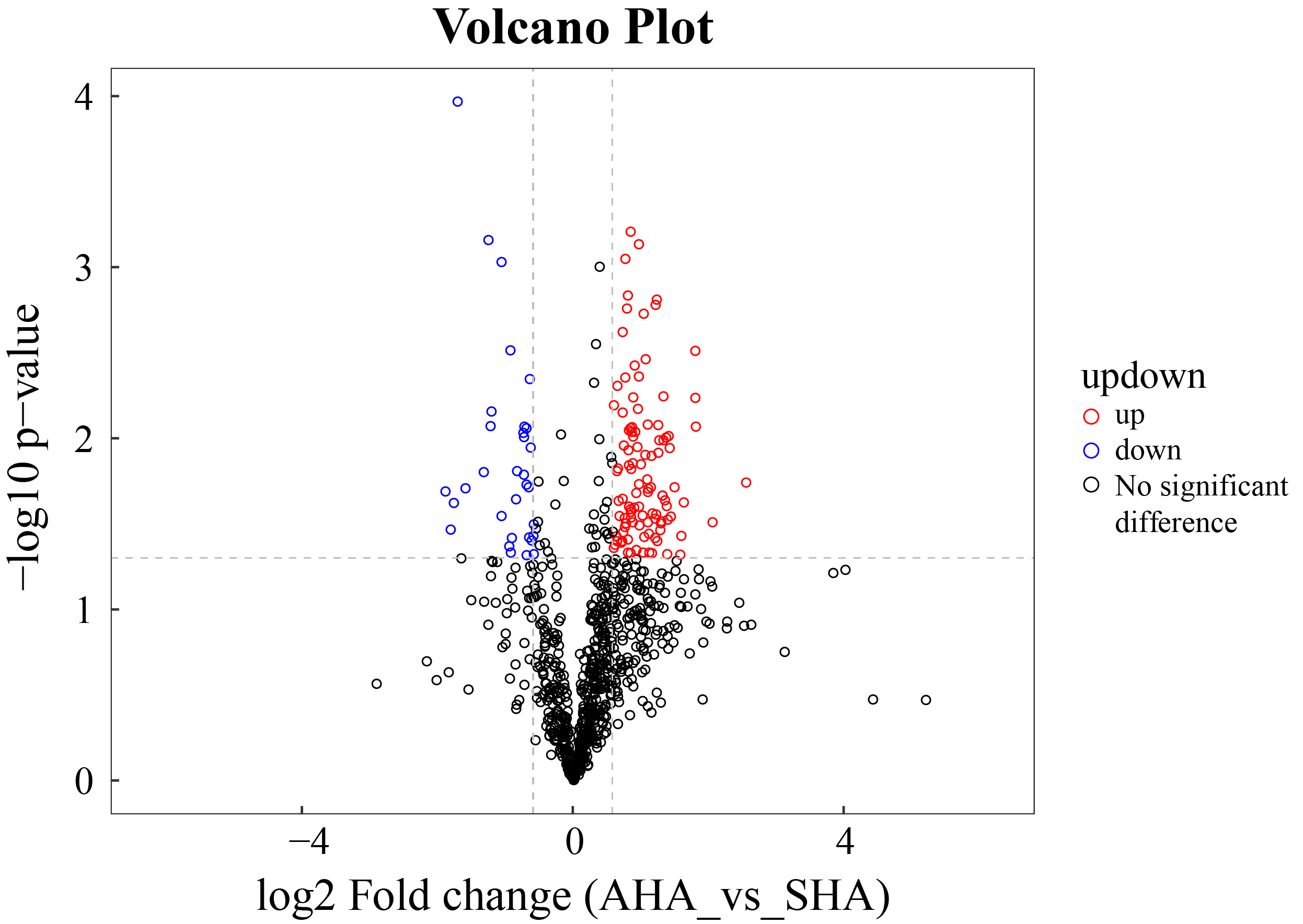

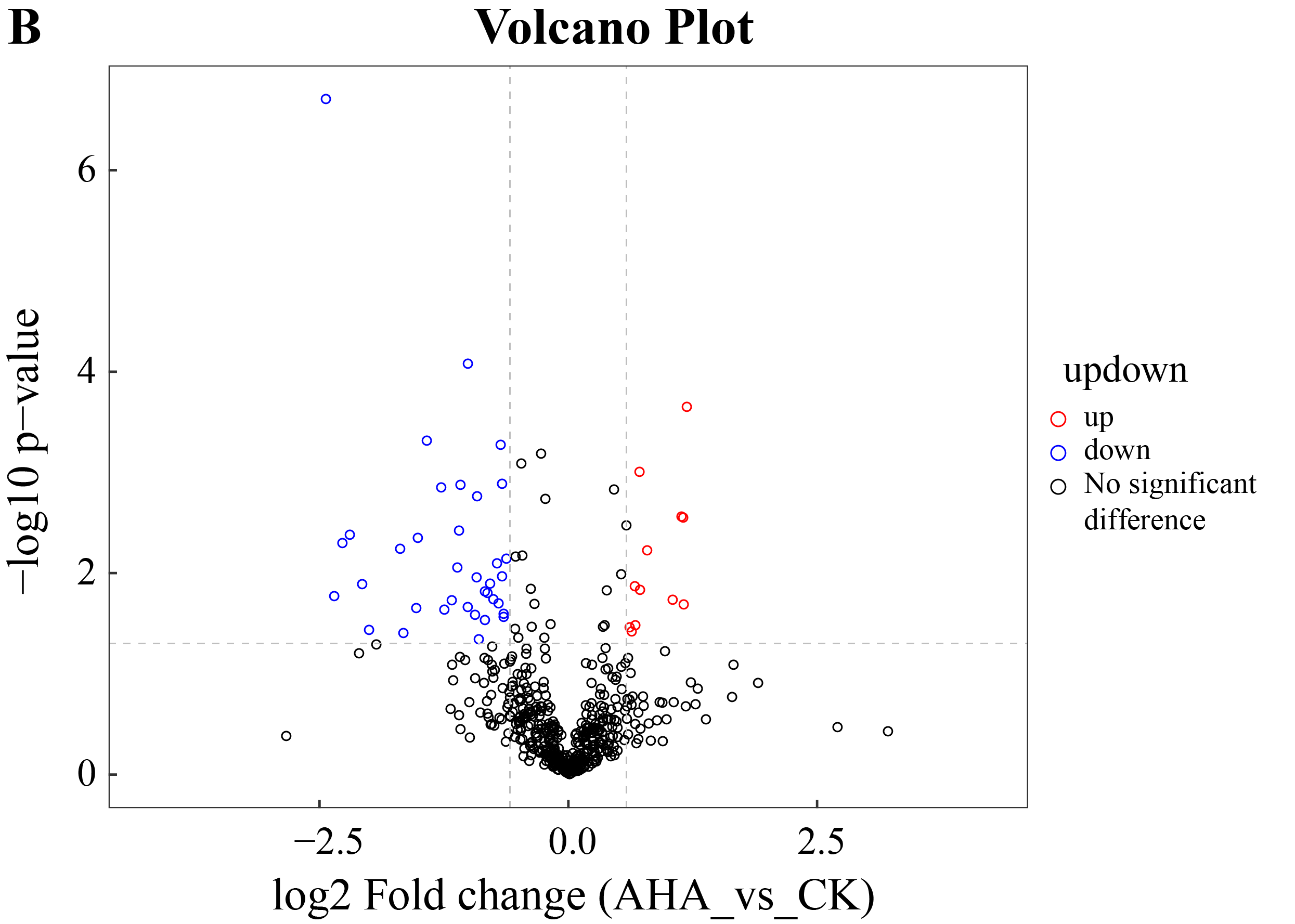

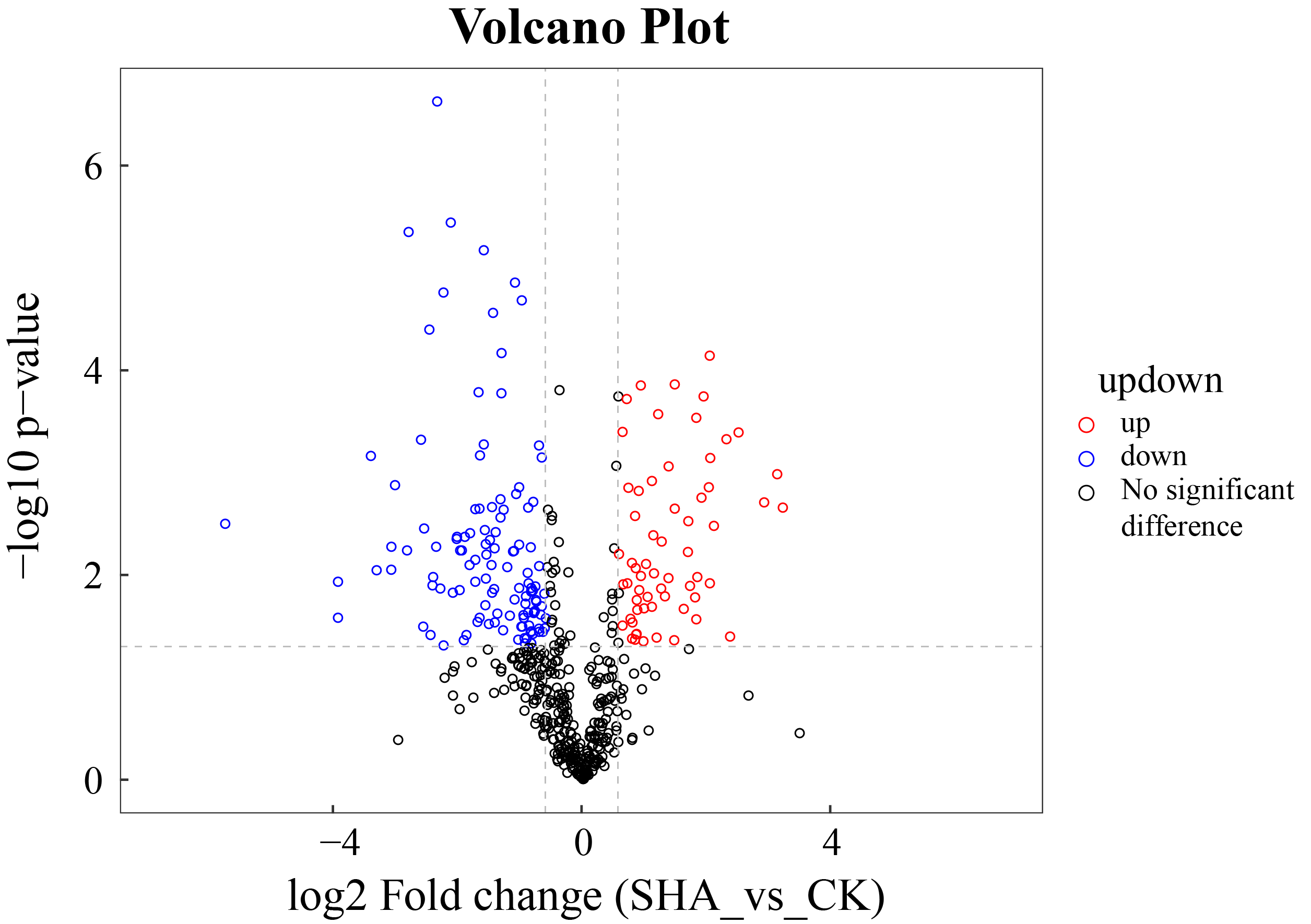

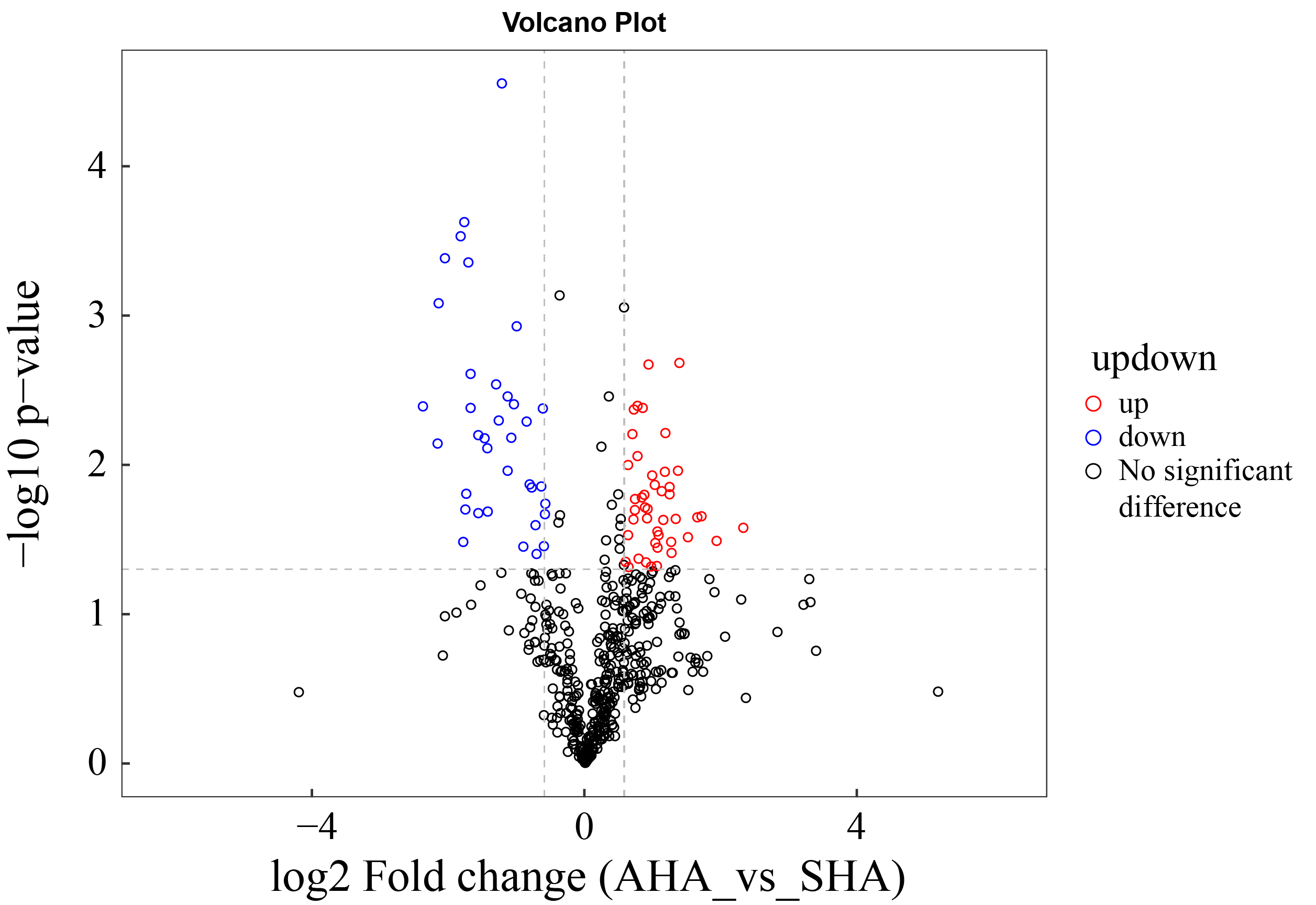


Fig. S4 Volcano-plot of the differential metabolites in ESI^+^ mode (A) and ESI^−^ mode (B) in response to different groups. The differential metabolites that up-regulated and down-regulated in abundance were indicated in red and blue, respectively.

Tab. S1. Metabolite information and VIP of each metabolite in Maize leaf.

| Compound | RT (min) | m/z | Formula | VIP  AHA/CK | VIP  SHA/CK | VIP  AHA/SHA | FC  AHA/CK | FC  SHA/CK | FC  AHA/SHA |
| --- | --- | --- | --- | --- | --- | --- | --- | --- | --- |
| [3-Hydroxy-1-(4-methoxy-7-oxofuro[3,2-g]chromen-9-yl)oxy-3-methylbutan-2-yl] (E)-2-methylbut-2-enoate | 0.649 | 455.11646 | C22H24O8 | 1.228 | 1.724 | 2.216 | 0.993 | 0.796* | 1.246* |
| 12-(((R)-2,3-Dihydroxypropyl)phosphoryloxy)-N,N,N-trimethylethanaminium | 0.667 | 258.10742 | [C8H21NO6P]+ | 0.398 | 1.236 | 1.280 | 1.766 | 6.232* | 0.283* |
| Palatinose | 0.682 | 360.14978 | C12H22O11 | 5.940 | 4.452 | 2.159 | 0.512** | 0.396** | 1.292** |
| Sucrose | 0.689 | 365.10263 | C12H22O11 | 1.931 | 1.443 | 0.754 | 0.593 ** | 0.488** | 1.214 |
| Carnitine | 0.7 | 162.1114 | C7H15NO3 | 0.541 | 1.030 | 1.038 | 1.572 | 3.293** | 0.477 |
| Cyromazine | 1.877 | 167.10332 | C6H10N6 | 2.559 | 3.072 | 3.904 | 1.022 | 0.444** | 2.303* |
| Loganin | 1.9 | 413.14169 |  | 1.364 | 1.622 | 1.378 | 0.765 | 0.434** | 1.760* |
| 2-(6-Aminopurin-9-yl)-5-(hydroxymethyl)oxolane-3,4-diol | 2.117 | 268.10416 | C10H13N5O4 | 2.960 | 2.560 | 1.372 | 0.765 | 0.681* | 1.123 |
| Cordycepin | 2.226 | 252.10828 | C10H13N5O3 | 1.091 | 1.576 | 1.364 | 0.835 | 0.599** | 1.394 |
| Phenmetrazine | 2.514 | 178.12279 | C11H15NO | 2.119 | 1.484 | 1.150 | 0.657* | 0.683* | 0.962 |
| Coumaric acid O-glucoside | 2.537 | 344.13553 | C15H18O8 | 1.082 | 0.960 | 0.534 | 0.568* | 0.453* | 1.254 |
| 3-O-Caffeoyl-4-O-methylquinic acid | 2.716 | 391.10034 | C17H20O9 | 4.483 | 3.408 | 1.491 | 0.204** | 0.042** | 4.805 |
| 2-beta-D-Glucopyranosyloxy-4-methoxy-cis-cinnamic acid | 2.839 | 374.147 | C16H20O9 | 1.875 | 1.558 | 0.770 | 0.495** | 0.371** | 1.335 |
| 6,7-Dimethoxy-8-(beta-D-glucopyranosyloxy)-2H-1-benzopyran-2-one | 2.878 | 407.07892 | C17H20O10 | 1.096 | 0.859 | 0.456 | 0.343** | 0.145** | 2.355* |
| 4-O-Feruloyl-D-quinic acid | 2.883 | 369.11703 | C17H20O9 | 2.189 | 1.774 | 0.941 | 0.314** | 0.101** | 3.093 |
| Khelloside | 2.891 | 409.10632 | C19H20O10 | 1.238 | 1.038 | 0.644 | 0.358* | 0.145** | 2.478 |
| Tryptophol | 3.137 | 144.0799 | C10H11NO | 0.349 | 1.328 | 1.584 | 1.147 | 0.071* | 16.141 |
| 2-[(2Z)-5-(Hexopyranosyloxy)-2-penten-1-yl]-3-oxo-, (1R,2R)-cyclopentaneacetic acid | 3.22 | 411.16058 | C18H28O9 | 1.454 | 1.614 | 2.058 | 0.908 | 0.425* | 2.137* |
| (15S,16Z,17S)-16-ethylidene-4-hydroxy-15-[(2S,3R,4S,5S,6R)-3,4,5-trihydroxy-6-(hydroxymethyl)oxan-2-yl]oxy-2,10,14,20-tetraoxatetracyclo[21.2.2.13,7.012,17]octacosa-1(25),3,5,7(28),12,23,26-heptaene-11,19-dione | 3.813 | 651.18597 | C32H36O13 | 0.182 | 0.973 | 1.105 | 1.022 | 1.827* | 0.559* |
| Murrayone | 3.918 | 259.09494 | C15H14O4 | 2.400 | 2.079 | 1.353 | 0.438** | 0.170** | 2.571** |
| Oxyterracin | 3.958 | 461.15454 | C22H24N2O9 | 3.885 | 2.614 | 0.418 | 0.101** | 0.080** | 1.253** |
| Methylparaben | 3.99 | 153.05331 | C8H8O3 | 0.711 | 1.138 | 1.827 | 0.751 | 1.359 | 0.553* |
| Methyl 2-[(1R,5R,6R,13S,14S,16S)-14-acetyloxy-6-(furan-3-yl)-1,5,15,15-tetramethyl-8,17-dioxo-7-oxatetracyclo[11.3.1.02,11.05,10]heptadec-10-en-16-yl]acetate | 4.129 | 535.23541 | C29H36O8 | 0.567 | 0.716 | 1.023 | 1.049 | 0.637* | 1.646** |
| HDMBOA-Glc | 4.352 | 388.11914 | C16H21NO10 | 1.098 | 0.637 | 0.909 | 0.438** | 0.788 | 0.316 |
| Sinapaldehyde | 4.462 | 209.07954 | C11H12O4 | 1.407 | 1.437 | 0.832 | 0.101** | 0.721** | 1.130 |
| Isoscoparin | 4.624 | 463.11996 | C22H22O11 | 0.424 | 0.716 | 1.018 | 1.082 | 0.452** | 2.394 |
| Verrucarol | 4.694 | 267.15814 | C15H22O4 | 1.449 | 1.519 | 1.216 | 0.655 | 0.349** | 1.878 |
| Rosiridoside B | 4.896 | 482.25366 | C21H36O11 | 2.142 | 1.990 | 1.441 | 0.632 | 0.470* | 1.345 |
| (4E,6E)-2-(Hydroxymethyl)-3-(3-methoxy-3-oxopropyl)deca-4,6-dienoic acid | 5.046 | 285.16785 | C15H24O5 | 1.924 | 2.770 | 2.612 | 0.830 | 0.423* | 1.963 |
| Meperidine | 5.073 | 248.16238 | C15H21NO2 | 1.178 | 0.878 | 0.405 | 0.471* | 0.419* | 1.124 |
| Ligustilide | 5.156 | 191.1041 | C12H14O2 | 1.129 | 0.403 | 0.989 | 0.751* | 1.058 | 0.710 |
| Hirsuteine | 5.755 | 367.2059 | C22H26N2O3 | 1.066 | 0.866 | 0.855 | 1.601 * | 1.701 | 0.941 |
| 3-[4-Methyl-1-(2-methylpropanoyl)-3-oxocyclohexyl]butanoic acid | 6.323 | 269.1763 | C15H24O4 | 0.149 | 1.254 | 1.594 | 1.036 | 0.299** | 3.468** |
| Puberanidine | 6.404 | 543.30688 | C30H42N2O7 | 0.830 | 1.179 | 1.015 | 0.738 | 0.300** | 2.464** |
| Brefeldin A | 6.724 | 281.17148 | C16H24O4 | 0.526 | 0.837 | 1.002 | 1.215 | 0.489* | 2.485* |
| 5-(6-Methyl-6-hydroxyheptyl)furan-2(5H)-one | 6.781 | 195.13808 | C12H20O3 | 0.766 | 3.705 | 4.631 | 1.046 | 0.404** | 2.590* |
| Trinoranastreptene | 7.023 | 161.13174 | C12H16 | 1.203 | 1.592 | 2.297 | 1.184 | 0.643* | 1.841** |
| 2-(1-Phenylethyl)-3,6-Dihydro-2H-Pyran | 7.023 | 189.12238 | C13H16O | 1.564 | 2.471 | 3.424 | 1.154 | 0.638* | 1.808** |
| 5-(6-Methyl-7-oxooctyl)furan-2(5H)-one | 7.025 | 247.13165 | C13H20O3 | 2.886 | 3.020 | 4.805 | 1.235 | 0.708* | 1.746* |
| 12-Hydroxy-13-(hydroxymethyl)-3,5,7-trimethyl-2,4-tetradecadienedioic acid | 7.194 | 271.16928 | C18H30O6 | 1.051 | 1.351 | 1.119 | 0.799 | 0.536* | 1.489 |
| (5S,6E,8S,9Z,13S,14R)-5-Hydroxy-8-methoxy-5,9,13,14-tetramethyl-1-oxacyclotetradeca-6,9-dien-2-one | 7.309 | 311.21945 | C18H30O4 | 2.317 | 3.812 | 3.507 | 1.318 | 2.139** | 0.616** |
| Lovastatin acid | 7.759 | 445.25696 | C24H38O6 | 0.711 | 1.375 | 1.447 | 0.800 | 0.259** | 3.084* |
| Vinpocetine | 7.784 | 351.21304 | C22H26N2O2 | 1.210 | 1.157 | 0.849 | 1.384* | 1.518 * | 0.912 |
| Isomuronic acid | 7.823 | 367.24326 | C21H34O5 | 1.160 | 1.835 | 1.804 | 0.787 | 0.337** | 2.337** |
| Dihydromuronic acid | 7.936 | 369.26062 | C21H36O5 | 1.520 | 0.439 | 1.139 | 1.389* | 1.044 | 1.330 |
| Cinobufagin | 8.141 | 443.23563 | C26H34O6 | 0.527 | 1.014 | 1.001 | 0.814 | 0.417* | 1.952* |
| Dehydrocholic acid | 8.142 | 403.24133 | C24H34O5 | 0.750 | 1.319 | 1.284 | 0.778 | 0.351** | 2.215* |
| N-(2-(cyclohexylamino)-2-oxo-1-(pyridin-3-yl)ethyl)-N-(furan-2-ylmethyl)-4-methylpentanamide | 8.152 | 412.26276 | C24H33N3O3 | 1.164 | 0.890 | 0.467 | 1.835* | 1.952** | 0.940 |
| Trimethoprim | 8.229 | 291.15048 | C14H18N4O3 | 1.478 | 1.429 | 1.027 | 0.585* | 0.309** | 1.894 |
| (8)-Shogaol | 8.231 | 305.20984 | C19H28O3 | 2.323 | 2.301 | 1.685 | 0.623 | 0.370** | 1.683* |
| 9-Oxooctadeca-10,12-dienoic acid | 8.369 | 295.22842 | C18H30O3 | 4.188 | 5.897 | 4.868 | 1.564 | 2.359** | 0.663* |
| Dihydroxyoctadecadienoic acid | 8.37 | 313.23825 | C18H32O4 | 1.447 | 2.307 | 2.096 | 1.404 | 2.184** | 0.643* |
| Safranal | 8.504 | 151.10979 |  | 1.428 | 1.672 | 1.470 | 0.773 | 0.458** | 1.687* |
| Kahweol | 8.573 | 315.19394 | C20H26O3 | 1.812 | 2.548 | 2.425 | 1.435 | 2.218** | 0.647 |
| (5beta,8alpha,9beta,10alpha,13alpha)-18-Methoxy-18-oxokauran-17-oic acid | 8.581 | 349.23563 | C21H32O4 | 0.231 | 1.602 | 1.727 | 0.929 | 0.225** | 4.136* |
| (6E)-Heptadeca-6,16-diene-1,2,4-triol | 8.643 | 307.22348 | C17H32O3 | 1.509 | 1.295 | 0.751 | 0.672 | 0.577* | 1.163 |
| 3-(2,5-Octadienyl)-oxiraneoctanoic acid | 8.657 | 277.21677 | C18H30O3 | 2.740 | 3.621 | 3.561 | 0.922 | 0.656 | 1.405* |
| 6,7-Diketolithocholic acid | 8.917 | 405.25967 | C24H36O5 | 0.809 | 0.676 | 1.161 | 1.550 | 0.447* | 3.464** |
| (1R,2R,4aS,8aS)-2-[(2R)-2-Hydroxybutyl]-1,3-dimethyl-1,2,4a,5,6,7,8,8a-octahydro-1-naphthalenecarboxylic acid | 8.997 | 263.19705 | C17H28O3 | 1.616 | 1.123 | 2.123 | 1.887 | 0.525** | 3.592 |
| Pergolide | 9.05 | 315.18991 | C19H26N2S | 1.097 | 2.418 | 3.276 | 1.068 | 0.584* | 1.828 |
| 3-[(4As,12aR)-2-methyl-1,3,4,5,12,12a-hexahydropyrido[3,4-b]acridin-4a-yl]phenol | 9.079 | 345.20346 | C23H24N2O | 1.292 | 0.565 | 0.778 | 0.349** | 0.666 | 0.524* |
| (E)-4-[(1R,2S,3S,4R,8As)-2,3,4-trihydroxy-2,5,5,8a-tetramethyl-3,4,4a,6,7,8-hexahydro-1H-naphthalen-1-yl]but-3-en-2-one | 9.158 | 328.24518 | C18H30O4 | 2.163 | 1.958 | 1.331 | 0.794 | 0.639* | 1.243 |
| 3a,4b,7a,12a-Tetrahydroxy-5b-cholanoic acid | 9.358 | 407.2796 | C24H40O6 | 0.534 | 1.204 | 1.582 | 1.257 | 0.262* | 4.789 |
| Andrastin D | 9.36 | 429.26123 | C26H36O5 | 0.593 | 1.069 | 1.495 | 1.365 | 0.222* | 6.143 |
| Isosteviol | 9.568 | 319.2222 | C20H30O3 | 1.135 | 2.410 | 3.017 | 1.035 | 0.536* | 1.931** |
| h_266_Oxandrolone_m3 | 9.604 | 305.20724 | C19H28O3 | 2.263 | 1.266 | 0.812 | 0.450** | 0.558* | 0.805 |
| 8-[3-Oxo-2-[(E)-pent-2-enyl]cyclopenten-1-yl]octanoic acid | 9.869 | 293.21078 | C18H28O3 | 2.671 | 2.118 | 1.326 | 1.189* | 1.173 | 1.014 |
| Glyceryl palmitate | 10.251 | 331.28333 | C19H38O4 | 0.593 | 1.256 | 1.430 | 0.894 | 0.514* | 1.740** |
| Fasciculic acid B | 10.355 | 659.40826 | C36H60O9 | 0.806 | 1.227 | 1.210 | 1.346 | 2.171* | 0.620 |
| 4-[5-[[4-[5-[Acetyl(hydroxy)amino]pentylamino]-4-oxobutanoyl]-hydroxyamino]pentylamino]-4-oxobutanoic acid | 10.365 | 478.2872 | C20H36N4O8 | 1.778 | 2.027 | 1.675 | 1.897 | 3.030** | 0.626 |
| Myxol (II) | 10.56 | 585.41486 | C40H56O3 | 1.514 | 0.924 | 1.851 | 1.280 | 0.776 | 1.649** |
| Linolenic acid | 10.836 | 279.22806 | C18H30O2 | 0.984 | 1.518 | 1.424 | 0.816 | 0.411** | 1.985 |
| [17-Acetyl-3-[5-[5-(3,5-dihydroxy-4-methoxy-6-methyloxan-2-yl)oxy-4-methoxy-6-methyloxan-2-yl]oxy-4-methoxy-6-methyloxan-2-yl]oxy-8,14,17-trihydroxy-10,13-dimethyl-1,2,3,4,5,6,7,9,11,12,15,16-dodecahydrocyclopenta[a]phenanthren-12-yl] benzoate | 10.838 | 935.51709 | C49H74O17 | 1.191 | 1.152 | 0.844 | 0.707 | 0.595* | 1.188 |
| Nandrolone | 10.868 | 275.19656 | C18H26O2 | 0.726 | 1.692 | 2.260 | 1.048 | 0.585 | 1.793** |
| Nostoxanthin | 11.093 | 601.42548 | C40H56O4 | 4.845 | 3.998 | 2.090 | 0.680* | 0.564** | 1.206 |
| Diadinochrome A | 11.099 | 583.41138 | C40H54O3 | 3.306 | 3.172 | 2.252 | 0.732* | 0.533** | 1.373* |
| 2-Palmitoylglycerol | 11.188 | 331.28583 | C19H38O4 | 4.801 | 4.798 | 7.492 | 0.925 | 1.056 | 0.876* |
| Peonidin-3,5-O-di-beta-glucopyranoside | 11.234 | 625.40143 | [C28H33O16]+ | 1.728 | 1.241 | 0.814 | 1.685* | 1.487* | 1.133 |
| h_61_17_Epioxandrolone | 11.332 | 307.22116 | C19H30O3 | 0.975 | 1.058 | 0.916 | 1.583 | 1.913* | 0.827 |
| Antheraxanthin | 11.449 | 585.42755 | C40H56O3 | 2.384 | 1.630 | 0.734 | 0.638* | 0.599** | 1.065 |
| (S)-7-(((2-O-6-Deoxy-alpha-L-mannopyranosyl)-beta-D-glucopyranosyl)oxy)-2,3-dihydro-5-hydroxy-2-(3-hydroxy-4-methoxyphenyl)-4H-1-benzopyran-4-one | 11.719 | 611.45581 | C28H34O15 | 1.639 | 1.991 | 1.718 | 0.816 | 0.591** | 1.380 |
| 6-(Diaminomethylideneamino)-2-[[(4E,6E,12E,14E)-3-hydroxy-2-(hydroxymethyl)-8,10,16-trimethyloctadeca-4,6,12,14-tetraenoyl]amino]hexanoic acid | 11.799 | 517.37939 | C29H50N4O5 | 0.890 | 1.017 | 0.919 | 0.698 | 0.262** | 2.670 |
| Diosmin | 11.981 | 609.40643 | C28H32O15 | 0.875 | 1.407 | 1.354 | 1.703 | 3.216** | 0.529 |
| PC 34:3\|PC 16:0_18:3 | 12.191 | 756.54156 | C42H78NO8P | 1.069 | 0.811 | 0.341 | 2.013* | 2.002 | 1.005 |
| 13'-Hydroxy-alpha-tocopherol | 12.241 | 429.36786 | C29H50O3 | 0.407 | 1.213 | 1.361 | 0.983 | 1.277** | 0.769 |
| Hupehenine | 12.637 | 416.35947 | C27H45NO2 | 0.333 | 1.172 | 1.370 | 1.326 | 4.355** | 0.305** |
| PC 32:4\|PC 14:1_18:3 | 12.659 | 726.51123 | C40H72NO8P | 1.681 | 0.767 | 1.611 | 0.563* | 0.935 | 0.602** |
| Dehydroeburicoic acid | 12.68 | 451.35754 | C31H48O3 | 1.904 | 2.907 | 2.486 | 0.765 | 0.381* | 2.007 |
| Pestalpolyol D | 12.69 | 566.47577 | C34H60O5 | 3.123 | 2.220 | 1.599 | 1.657* | 1.633* | 1.015 |
| 9s,13r-12-Oxophytodienoic Acid | 12.693 | 275.19836 | C18H28O3 | 3.004 | 2.500 | 1.813 | 1.529* | 1.694* | 0.903 |
| (3R,4R,5R)-3-[(11E)-11,15-Hexadecadien-9-yn-1-yl]-4-hydroxy-5-methyldihydro-2(3H)-furanone | 12.768 | 333.23798 | C21H32O3 | 1.361 | 1.682 | 1.418 | 0.880 | 0.714* | 1.232 |
| HexCer 34:3;2O\|HexCer 18:2;2O/16:1 | 12.78 | 696.53833 | C40H73NO8 | 0.416 | 2.809 | 3.617 | 0.997 | 1.579* | 0.632* |
| Cer 34:3;2O\|Cer 18:2;2O/16:1 | 12.781 | 534.48279 | C34H63NO3 | 0.620 | 2.150 | 2.630 | 1.044 | 1.535* | 0.680* |
| HexCer 34:2;3O\|HexCer 18:2;2O/16:0;O | 12.784 | 714.54205 | C40H75NO9 | 0.284 | 1.580 | 1.983 | 1.027 | 1.605* | 0.640 |
| 6-{9a,11a-dimethyl-1H,2H,3H,3aH,3bH,4H,8H,9H,9bH,10H,11H-cyclopenta[a]phenanthren-1-yl}-3-ethyl-2-methylheptane | 12.874 | 397.38248 | C29H48 | 2.577 | 2.582 | 2.229 | 1.186 | 1.302* | 0.911 |
| Cholestenone | 13.202 | 385.34335 | C27H44O | 1.148 | 1.023 | 0.779 | 1.376 | 1.623* | 0.848 |
| DG 36:6\|DG 18:3_18:3 | 13.262 | 630.50989 | C39H64O5 | 1.286 | 3.263 | 3.685 | 0.866 | 0.210* | 4.122 |
| 2,3-dimethyl-5-(3,7,11,15-tetramethylhexadec-2-en-1-yl)cyclohexa-2,5-diene-1,4-dione | 13.263 | 415.35202 | C28H46O2 | 0.639 | 1.290 | 1.399 | 2.303 | 8.562** | 0.269* |
| PC 36:4\|PC 18:2_18:2 | 13.314 | 782.56665 | C44H80NO8P | 0.819 | 1.259 | 1.245 | 2.462 | 5.759** | 0.428 |
| DG(18:2(9Z,12Z)/18:2(9Z,12Z)) | 13.427 | 599.49683 | C39H68O5 | 0.358 | 1.595 | 2.330 | 0.957 | 1.442 | 0.664* |
| Fecosterol | 13.46 | 399.358 | C28H46O | 1.520 | 2.217 | 2.206 | 1.383* | 2.170** | 0.637** |
| Stigmasterol | 13.751 | 413.37741 | C29H48O | 2.010 | 4.458 | 4.798 | 1.393 | 3.502** | 0.398* |
| Lupenone | 13.907 | 425.37433 | C30H48O | 0.616 | 0.835 | 1.137 | 1.172 | 2.269* | 0.516* |
| cis-Aconitic acid | 0.669 | 173.01089 | C6H6O6 | 1.789 | 1.019 | 1.390 | 0.820** | 0.774** | 1.060 |
| Trehalose | 0.686 | 341.11005 | C12H22O11 | 6.222 | 4.365 | 1.432 | 0.493** | 0.332** | 1.483** |
| 2-Isopropylmalic acid | 2.045 | 175.06081 | C7H12O5 | 1.719 | 1.172 | 0.630 | 0.441* | 0.285** | 1.547 |
| 3-[3-(3,4-Dihydroxyphenyl)prop-2-enoyloxy]-1,4,5-trihydroxycyclohexane-1-carboxylic acid | 2.106 | 353.08591 | C16H18O9 | 1.081 | 0.740 | 0.306 | 0.194* | 0.019** | 10.460 |
| 3-p-Coumaroylquinic acid | 2.587 | 337.09302 | C16H18O8 | 2.472 | 1.887 | 0.714 | 0.248* | 0.065* | 3.795* |
| (1r,3R,4s,5S)-4-{[(2E)-3-(3,4-Dihydroxyphenyl)-2-propenoyl]oxy}-1,3,5-trihydroxycyclohexanecarboxylic acid | 2.601 | 353.08844 | C16H18O9 | 1.120 | 0.712 | 0.174 | 0.217** | 0.123** | 1.755 |
| 5-O-Feruloylquinic acid | 2.882 | 367.10239 | C17H20O9 | 6.374 | 4.871 | 2.333 | 0.409** | 0.144** | 2.841* |
| 2,5-Dihydroxybenzoic acid | 3.018 | 329.02777 | C7H6O4 | 1.793 | 2.110 | 1.967 | 1.492 | 2.221* | 0.672 |
| 13:4+4O Fatty acyl hexoside | 3.298 | 415.16025 | C19H28O10 | 2.586 | 3.918 | 3.918 | 0.907 | 0.362** | 2.504* |
| HMBOA-Glc | 3.684 | 356.09592 | C15H19NO9 | 2.356 | 1.483 | 0.121 | 0.206** | 0.171** | 1.206 |
| DIMBOA-Glc | 3.744 | 372.0907 | C15H19NO10 | 2.764 | 1.916 | 0.587 | 0.23*6 | 0.119** | 1.988 |
| Flavone base + 3O, C-Pen, C-Pen | 4.54 | 533.13 | C25H26O13 | 0.905 | 1.625 | 1.666 | 1.233 | 1.986* | 0.621 |
| Kaempferol-3-O-glucoside | 5.055 | 447.09286 | C21H20O11 | 0.592 | 1.328 | 1.415 | 1.292 | 4.151** | 0.311** |
| 5,7-Dihydroxy-2-(4-hydroxy-3-methoxyphenyl)-3-[3,4,5-trihydroxy-6-[[(2R,3R,4R,5R,6S)-3,4,5-trihydroxy-6-methyloxan-2-yl]oxymethyl]oxan-2-yl]oxychromen-4-one | 5.107 | 623.15875 | C28H32O16 | 1.231 | 1.610 | 1.504 | 1.035 | 1.795* | 0.577 |
| 9-Hydroxy-11-[3-hydroxy-5-(1-hydroxypropyl)oxolan-2-yl]undec-10-enoic acid | 5.744 | 343.21286 | C18H32O6 | 1.712 | 0.866 | 0.633 | 2.182 ** | 1.575 | 1.385 |
| 13-Oxotrideca-9,11-dienoic acid | 7.02 | 223.13367 | C13H20O3 | 9.089 | 6.416 | 10.325 | 1.223 | 0.563* | 2.174* |
| 9,12,13-Trihydroxyoctadeca-10,15-dienoic acid | 7.311 | 327.21747 | C18H32O5 | 2.825 | 4.372 | 4.249 | 1.244 | 1.915** | 0.650** |
| 16-Hydroxy-9-oxooctadeca-10,12,14-trienoic acid | 7.495 | 307.19006 | C18H28O4 | 1.607 | 1.679 | 1.510 | 1.486** | 1.866** | 0.796 |
| 12,13-Dihydroxy-9-oxooctadeca-10,15-dienoic acid | 7.803 | 325.20203 | C18H30O5 | 2.180 | 3.531 | 3.599 | 1.390 | 2.793** | 0.498** |
| 9-Oxo-11-(3-pentyloxiran-2-YL)undec-10-enoic acid | 8.05 | 309.20569 | C18H30O4 | 4.533 | 4.451 | 3.694 | 0.708 | 0.336** | 2.108* |
| 8-(2-Oxo-5-pent-2-enylcyclopent-3-en-1-yl)octanoic acid | 8.05 | 291.19363 | C18H28O3 | 1.212 | 1.030 | 0.750 | 0.589 | 0.283** | 2.077* |
| Jasmonic acid | 8.224 | 209.11684 | C12H18O3 | 2.128 | 1.893 | 1.254 | 0.636 | 0.355* | 1.789 |
| 12-Hydroxy-5,8,10-heptadecatrienoic acid | 9.017 | 279.19647 | C17H28O3 | 3.024 | 1.476 | 2.750 | 2.406 | 0.247* | 9.759 |
| 3-Dehydrocholic acid | 9.358 | 405.2627 | C24H38O5 | 1.083 | 1.074 | 1.295 | 1.332 | 0.189* | 7.055 |
| 13-HoTrE | 9.435 | 293.21127 | C18H30O3 | 1.955 | 3.313 | 3.433 | 0.902 | 0.544* | 1.660* |
| [2-Hydroxy-3-[3,4,5-trihydroxy-6-[[3,4,5-trihydroxy-6-(hydroxymethyl)oxan-2-yl]oxymethyl]oxan-2-yl]oxypropyl] octadeca-9,12,15-trienoate | 9.78 | 675.35455 | C33H56O14 | 1.460 | 0.795 | 0.555 | 0.625* | 0.547* | 1.143 |
| 3-(Hexopyranosyloxy)-2-hydroxypropyl ester, (9Z,12Z,15Z)-9,12,15-octadecatrienoic acid | 10.058 | 559.30774 | C27H46O9 | 1.852 | 1.116 | 0.710 | 0.632* | 0.529* | 1.196 |
| LPG 16:1 | 10.133 | 481.25534 | C22H43O9P | 4.850 | 7.637 | 7.593 | 0.896 | 0.492** | 1.821* |
| 9-Hydroxy-10E,12Z-octadecadienoic acid | 10.272 | 295.2262 | C18H32O3 | 0.625 | 1.036 | 1.046 | 1.552 | 3.858** | 0.402** |
| [3-[2-Aminoethoxy(hydroxy)phosphoryl]oxy-2-hydroxypropyl] octadeca-9,12-dienoate | 10.365 | 476.2724 | C23H44NO7P | 0.859 | 1.077 | 0.964 | 1.946 | 3.251** | 0.599 |
| FA 16:0;2O | 10.535 | 271.22885 | C16H32O3 | 2.929 | 4.353 | 4.436 | 1.670 | 3.552** | 0.470** |
| Euscaphic acid | 10.611 | 487.34006 |  | 1.561 | 1.483 | 1.828 | 1.397 | 0.141** | 9.886 |
| Ginkgolic acid C17-1 | 10.616 | 373.27261 | C24H38O3 | 1.347 | 1.495 | 1.381 | 0.760 | 0.204* | 3.716 |
| Ginsenoside-Rg1 | 11.627 | 845.46954 | C42H72O14 | 0.952 | 1.026 | 0.630 | 0.664 | 0.316** | 2.101 |
| Stearic acid | 11.94 | 283.26343 | C18H36O2 | 3.088 | 5.407 | 5.917 | 1.063 | 1.879* | 0.566* |
| PE 36:4 | 12.331 | 738.50665 | C41H74NO8P | 0.800 | 1.054 | 0.853 | 1.377 | 2.027** | 0.679 |
| PMeOH 34:3\|PMeOH 16:0_18:3 | 12.928 | 683.46027 | C38H69O8P | 2.618 | 2.271 | 3.310 | 0.555* | 1.300 | 0.427** |
| PI 34:2 | 12.936 | 833.51678 | C43H79O13P | 0.721 | 1.441 | 1.534 | 0.931 | 2.601* | 0.358** |
| PMeOH 36:4\|PMeOH 18:2_18:2 | 12.953 | 709.48108 | C40H71O8P | 1.088 | 1.697 | 2.011 | 0.674 | 2.167* | 0.311** |
| PMeOH 34:2\|PMeOH 16:0_18:2 | 13.252 | 685.48163 | C38H71O8P | 2.327 | 3.155 | 3.909 | 0.579 | 2.062* | 0.281** |
| PG 34:1\|PG 16:0_18:1 | 13.377 | 747.51837 | C40H77O10P | 0.415 | 1.013 | 1.082 | 1.191 | 4.964** | 0.240** |
| PMeOH 34:1\|PMeOH 16:0_18:1 | 13.688 | 687.49567 | C38H73O8P | 0.577 | 1.020 | 1.169 | 0.705 | 2.416** | 0.292** |
| trans-Zeatin | 3.812 | 218.10213 | C10H13N5O | 0.202 | 0.178 | 0.135 | 1.579* | 1.562** | 1.011 |
| Zeatin riboside | 4.066 | 352.15808 | C15H21N5O5 | 0.160 | 0.329 | 0.369 | 0.901 | 0.441** | 2.044** |
| Methyl jasmonate | 6.689 | 225.14542 | C13H20O3 | 0.974 | 0.655 | 0.578 | 0.414** | 0.440** | 0.871 |
| 12-Hydroxyjasmonic acid | 3.651 | 209.11397 | C12H18O4 | 0.368 | 0.678 | 0.678 | 0.997 | 0.512** | 0.512** |

Tab. S2. Analysis of metabolic pathways.

| comparison | metabolic pathway | Test | TestAll | Ref | RefAll | Test_per | Ref_per | p.value | FDR | richFactor | match compound |
| --- | --- | --- | --- | --- | --- | --- | --- | --- | --- | --- | --- |
| AHA-CK | Starch and sucrose metabolism | 2 | 9 | 37 | 4746 | 22.222 | 0.780 | 0.002 | 0.029 | 0.054 | Trehalose\| Sucrose |
|  | ABC transporters | 2 | 9 | 137 | 4746 | 22.222 | 2.887 | 0.026 | 0.163 | 0.015 | Trehalose \|Sucrose |
|  | Citrate cycle (TCA cycle) | 1 | 9 | 20 | 4746 | 11.111 | 0.421 | 0.037 | 0.163 | 0.050 | cis-Aconitic acid |
|  | Stilbenoid, diarylheptanoid and gingerol biosynthesis | 1 | 9 | 25 | 4746 | 11.111 | 0.527 | 0.046 | 0.163 | 0.040 | 3-p-Coumaroylquinic acid |
|  | C5-Branched dibasic acid metabolism | 1 | 9 | 34 | 4746 | 11.111 | 0.716 | 0.063 | 0.176 | 0.029 | cis-Aconitic acid |
|  | Galactose metabolism | 1 | 9 | 46 | 4746 | 11.111 | 0.969 | 0.084 | 0.185 | 0.022 | Sucrose |
|  | Glyoxylate and dicarboxylate metabolism | 1 | 9 | 62 | 4746 | 11.111 | 1.306 | 0.112 | 0.185 | 0.016 | cis-Aconitic acid |
|  | Anthocyanin biosynthesis | 1 | 9 | 66 | 4746 | 11.111 | 1.391 | 0.119 | 0.185 | 0.015 | Peonidin-3,5-O-di-beta-glucopyranoside |
|  | Phenylpropanoid biosynthesis | 1 | 9 | 68 | 4746 | 11.111 | 1.433 | 0.122 | 0.185 | 0.015 | 3-p-Coumaroylquinic acid |
|  | Flavonoid biosynthesis | 1 | 9 | 74 | 4746 | 11.111 | 1.559 | 0.132 | 0.185 | 0.014 | 3-p-Coumaroylquinic acid |
|  | Carotenoid biosynthesis | 1 | 9 | 115 | 4746 | 11.111 | 2.423 | 0.198 | 0.252 | 0.009 | 3-p-Coumaroylquinic acid |
|  | 2-Oxocarboxylic acid metabolism | 1 | 9 | 134 | 4746 | 11.111 | 2.823 | 0.227 | 0.265 | 0.007 | cis-Aconitic acid |
|  | Biosynthesis of secondary metabolites | 4 | 9 | 2059 | 4746 | 44.444 | 43.384 | 0.601 | 0.647 | 0.002 | Trehalose\| cis-Aconitic acid\| 3-p-Coumaroylquinic acid\| Sucrose |
|  | Metabolic pathways | 3 | 9 | 2764 | 4746 | 33.333 | 58.239 | 0.968 | 0.968 | 0.001 | Trehalose\| cis-Aconitic acid\| Sucrose |
| SHA-CK | Starch and sucrose metabolism | 2 | 17 | 37 | 4746 | 11.765 | 0.780 | 0.007 | 0.142 | 0.054 | Trehalose\| Sucrose |
|  | Biosynthesis of unsaturated fatty acids | 2 | 17 | 74 | 4746 | 11.765 | 1.559 | 0.028 | 0.266 | 0.027 | Stearic acid \|Linolenic acid |
|  | Citrate cycle (TCA cycle) | 1 | 17 | 20 | 4746 | 5.882 | 0.421 | 0.069 | 0.327 | 0.050 | cis-Aconitic acid |
|  | ABC transporters | 2 | 17 | 137 | 4746 | 11.765 | 2.887 | 0.085 | 0.327 | 0.015 | Trehalose\| Sucrose |
|  | Stilbenoid, diarylheptanoid and gingerol biosynthesis | 1 | 17 | 25 | 4746 | 5.882 | 0.527 | 0.086 | 0.327 | 0.040 | 3-p-Coumaroylquinic acid |
|  | C5-Branched dibasic acid metabolism | 1 | 17 | 34 | 4746 | 5.882 | 0.716 | 0.115 | 0.329 | 0.029 | cis-Aconitic acid |
|  | alpha-Linolenic acid metabolism | 1 | 17 | 44 | 4746 | 5.882 | 0.927 | 0.147 | 0.329 | 0.023 | Linolenic acid |
|  | Galactose metabolism | 1 | 17 | 46 | 4746 | 5.882 | 0.969 | 0.153 | 0.329 | 0.022 | Sucrose |
|  | Fatty acid biosynthesis | 1 | 17 | 58 | 4746 | 5.882 | 1.222 | 0.189 | 0.329 | 0.017 | Stearic acid |
|  | Glyoxylate and dicarboxylate metabolism | 1 | 17 | 62 | 4746 | 5.882 | 1.306 | 0.201 | 0.329 | 0.016 | cis-Aconitic acid |
|  | Anthocyanin biosynthesis | 1 | 17 | 66 | 4746 | 5.882 | 1.391 | 0.212 | 0.329 | 0.015 | Peonidin-3,5-O-di-beta-glucopyranoside |
|  | Phenylpropanoid biosynthesis | 1 | 17 | 68 | 4746 | 5.882 | 1.433 | 0.218 | 0.329 | 0.015 | 3-p-Coumaroylquinic acid |
|  | Flavonoid biosynthesis | 1 | 17 | 74 | 4746 | 5.882 | 1.559 | 0.235 | 0.329 | 0.014 | 3-p-Coumaroylquinic acid |
|  | Tyrosine metabolism | 1 | 17 | 78 | 4746 | 5.882 | 1.643 | 0.246 | 0.329 | 0.013 | 2,5-Dihydroxybenzoic acid |
|  | Tryptophan metabolism | 1 | 17 | 83 | 4746 | 5.882 | 1.749 | 0.260 | 0.329 | 0.012 | Tryptophol |
|  | Carotenoid biosynthesis | 1 | 17 | 115 | 4746 | 5.882 | 2.423 | 0.341 | 0.405 | 0.009 | Nostoxanthin |
|  | 2-Oxocarboxylic acid metabolism | 1 | 17 | 134 | 4746 | 5.882 | 2.823 | 0.386 | 0.431 | 0.007 | cis-Aconitic acid |
|  | Biosynthesis of secondary metabolites | 5 | 17 | 2059 | 4746 | 29.412 | 43.384 | 0.923 | 0.975 | 0.002 | Trehalose\| cis-Aconitic acid\| 3-p-Coumaroylquinic acid\| Sucrose \| Linolenic acid |
|  | Metabolic pathways | 5 | 17 | 2764 | 4746 | 29.412 | 58.239 | 0.996 | 0.996 | 0.002 | Trehalose\| cis-Aconitic acid\| 2,5-Dihydroxybenzoic acid\|Sucrose\|Linolenic acid |
| AHA-SHA | Starch and sucrose metabolism | 1 | 6 | 37 | 4746 | 16.667 | 0.780 | 0.046 | 0.180 | 0.027 | Trehalose |
|  | Fatty acid biosynthesis | 1 | 6 | 58 | 4746 | 16.667 | 1.222 | 0.071 | 0.180 | 0.017 | Stearic acid |
|  | Biosynthesis of unsaturated fatty acids | 1 | 6 | 74 | 4746 | 16.667 | 1.559 | 0.090 | 0.180 | 0.013 | Stearic acid |
|  | ABC transporters | 1 | 6 | 137 | 4746 | 16.667 | 2.887 | 0.161 | 0.242 | 0.007 | Trehalose |
|  | Biosynthesis of secondary metabolites | 1 | 6 | 2059 | 4746 | 16.667 | 43.384 | 0.967 | 0.995 | 0.0005 | Trehalose |
|  | Metabolic pathways | 1 | 6 | 2764 | 4746 | 16.667 | 58.239 | 0.995 | 0.995 | 0.0004 | Trehalose |
